# Intravitreal injection of Huperzine A promotes retinal ganglion cells survival and axonal regeneration after optic nerve crush

**DOI:** 10.3389/fncel.2023.1145574

**Published:** 2023-05-24

**Authors:** Lai-Yang Zhou, Di Chen, Xin-Ran Guo, Yu-Qian Niu, Yong-Sai Xu, Dong-Fu Feng, Tie-Chen Li

**Affiliations:** ^1^School of Preclinical Medicine, Wannan Medical College, Wuhu, China; ^2^Department of Neurosurgery, Shanghai Jiao Tong University Affiliated Sixth People’s Hospital South Campus, Shanghai, China; ^3^Department of Neurosurgery, The First Affiliated Hospital of Zhengzhou University, Zhengzhou, China; ^4^Fengxian District Central Hospital Graduate Student Training Base, Jinzhou Medical University, Shanghai, China; ^5^School of Medicine, Anhui University of Science and Technology, Huainan, China

**Keywords:** traumatic optic neuropathy (TON), Huperzine A (HupA), retinal ganglion cell (RGC), axonal regeneration, survival, mTOR

## Abstract

Traumatic optic neuropathy (TON) is a condition that causes massive loss of retinal ganglion cells (RGCs) and their axonal fibers, leading to visual insufficiency. Several intrinsic and external factors can limit the regenerative ability of RGC after TON, subsequently resulting in RGC death. Hence, it is important to investigate a potential drug that can protect RGC after TON and enhance its regenerative capacity. Herein, we investigated whether Huperzine A (HupA), extracted from a Chinese herb, has neuroprotective effects and may enhance neuronal regeneration following the optic nerve crush (ONC) model. We compared the three modes of drug delivery and found that intravitreal injection of HupA could promote RGC survival and axonal regeneration after ONC. Mechanistically, HupA exerted its neuroprotective and axonal regenerative effects through the mTOR pathway; these effects could be blocked by rapamycin. To sum up, our findings suggest a promising application of HupA in the clinical treatment of traumatic optic nerve.

## Introduction

Traumatic optic neuropathy (TON) is one of the important causes of blindness in the world, resulting in axonal rupture and retinal ganglion cell (RGC) apoptosis (Li et al., 2017), which significantly reduces the patients’ quality of life. TON often affect young population (Lee et al., 2010). The major goal of treatment after TON is to maintain the survival of damaged neurons and enhance their regenerative capacity (Varadarajan et al., 2022). However, most adult mammalian RGCs lose their intrinsic regenerative ability and gradually undergo apoptosis after TON (Chen D. et al., 2022). Corticosteroid or surgical decompression has been widely used as the main therapeutic method, but the clinical outcome of TON remains unsatisfactory (Wladis et al., 2021; Chen B. et al., 2022).

Several natural products have been shown to possess neuroprotective properties (Bandiwadekar et al., 2022). For example, *Ginkgo biloba* and *Lycium barbarum* can reduce RGC apoptosis after optic nerve injury (Xu et al., 2014). In addition, Huperzine A (HupA), an extract of a Chinese herb that was initially described as an acetylcholinesterase inhibitor (AChEI) (Mao et al., 2016), is used to treat cognitive disorders including Alzheimer’s disease and other forms of dementia (Damar et al., 2016). *In vitro* experiments revealed that HupA might have neuroprotective effects by reducing neurotoxicity, apoptosis, and inflammation (Friedli and Inestrosa, 2021; Shukla et al., 2022). However, the role of HupA on neurotrauma, especially on TON, has not been described.

In the present study, we explored the ability of HupA to protect RGC and promote optic nerve regeneration after optic nerve crush (ONC). We compared the efficiency of different administration routes of HupA on RGC and found that intravitreal injection of HupA may significantly promote survival and axonal regeneration of RGC after ONC. Furthermore, we demonstrated that HupA could enhance mTOR activity to achieve its biological effects.

## Materials and methods

### Animals

6-week-old C57BL/6 mice were obtained from SPF Biotechnology, Beijing, China. All animal procedures were authorized by the Committee of Shanghai Sixth People’s Hospital affiliated with Shanghai Jiao Tong University (approval number: 2022-0333). Animals were maintained in a 12-h light/dark cycle at a temperature of 20–24°C and provided food and water *at libitum*.

### Drug handling and administration

**FIGURE 1:**
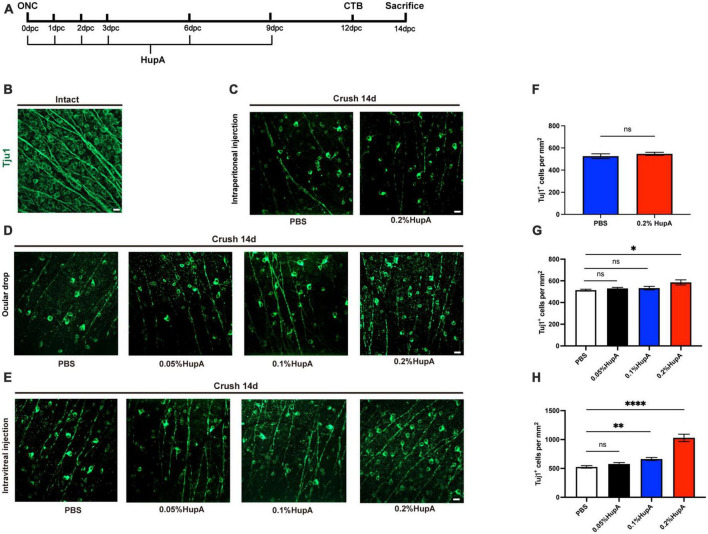
Ocular administration of HupA promotes RGCs survival after optic nerve crush and the effect of different doses on RGCs survival with HupA-treatment. **(A)** Experimental timeline for HupA administration. Crush, optic nerve crush; HupA, intravitreal injection of HupA; CTB, intravitreal injection of CTB-555; d: 14 days post-crush. **(B)** Representative confocal image of wholemount intact retinas showing Tuj1-labeled RGCs (green). Scale bar, 20 mm. **(C)** Representative confocal image of retinal whole mounts shows the surviving Tuj1-labeled RGCs (green) at 14dpc with intraperitoneal injection of PBS/HupA. Scale bar, 20 mm. **(D)** Representative confocal image of retinal whole mounts shows the surviving Tuj1-labeled RGCs (green) at 14dpc with ocular drip of PBS, 0.05% HupA, 0.1% HupA, and 0.2% HupA, respectively. Scale bar, 20 mm. **(E)** Representative confocal image of retinal whole mounts shows the surviving Tuj1-labeled RGCs (green) at 14dpc with intravitreal injection of PBS, 0.05% HupA, 0.1% HupA, and 0.2% HupA, respectively. Scale bar, 20 mm. **(F–H)** Quantification of surviving RGCs with each group in **(A–C)**, respectively (Data are represented as mean ± SEM, unpaired *t*-test, *n* = 6 in each group, *p < 0.05, ^**^p < 0.01, ^****^p < 0.0001).

The main ingredient of Huperzine A injection (Wepon Pharmaceutical Group Co., Ltd., China) is 0.2% Huperzine A (0.2 mg/ml). Before administration, the HupA and phosphate buffer solution (PBS) were diluted in hydrochloric acid, sodium chloride, and water, respectively. Based on the effective dose of HupA administered intraperitoneally in previous studies (Mei et al., 2021), 0.5 mg/kg of HupA was administered once a day for 14 days after ONC. Ocular drops of 0.2% HupA were administered thrice daily for 14 days after ONC. Intravitreal injection of 1 μl of 0.2% HupA was administered 6 times after ONC (Figure 1A). We used the solvent from the HupA injection to dilute 1 × PBS (phosphate-buffered saline), and PBS diluted at the same ratio as the control injection. In each of the different administration mode experiments, the control group was administered diluted PBS injection.

Rapamycin administration was used as previously described (Chen D. et al., 2022). Ethanol was used to dilute rapamycin (Selleckchem) to 20 mg/ml. Rapamycin was diluted in PBS with 5% Tween 80 and 5% polyethylene glycol 400 (0.5–1.5 mg/ml) before injection. A total of 6 mg/kg rapamycin or an equivalent amount of PBS was then intraperitoneally injected every 2 days after ONC.

### Optic nerve crush (ONC) model and anterograde labeling

Optic nerve crush (ONC) model, axonal labeling, intravitreal injection, and RGC survival analysis were performed as reported in previous studies (Park et al., 2008; Chen D. et al., 2022). Before ONC, mice were anesthetized with 2% sodium pentobarbital (100 mg/kg). ONC was performed in the left eye of all experimental mice. Fine surgical forceps were used for crushing the exposed intraorbital optic nerve at approximately 0.5–1.0 mm from the posterior pole of the eye for 5 s. Aureomycin eye ointment was applied to the left eye to prevent infection after ONC. Twelve days after ONC, 1.5 μl of CTB-555 [Cholera Toxin Subunit B (Recombinant)-Alexa Fluor™ 555 Conjugate, Thermo Fisher Scientific, C34776] was injected into the left vitreous humor using WPI Nanofl syringe (35-gauge needle). Injection of CTB-555 can label the axons of RGC, enabling observation of axon growth after ONC.

### Intravitreal injection

Before intravitreal injection, mice were given 2% sodium pentobarbital (100 mg/kg) anesthesia. For intravitreal injection of drug or PBS, the injection needle was inserted into the eye of the 6-week-old mouse, behind the orthoptic lens, and into the vitreous chamber to prevent lens damage. In the HupA-injected mice, an intravitreal injection of 2 μl HupA was given to the left eye, while the right eye was left untreated. In the control group, 2 μl PBS was injected into the left eye using the same approach.

### Perfusions and tissue processing

Mice were administered an overdose of anesthetic, transcardially perfused with ice-cold PBS, and then given 4% paraformaldehyde (PFA, biosharp). After perfusion, the eyeball and optic nerve were dissected and fixed overnight at 4°C in 4% PFA. Tissues for sectioning were dehydrated through 30% sucrose at 4°C for 48 h. Next, the tissues were embedded in optimal cutting temperature compound (OCT) using cryostat for retinal or optic nerve sectioning. The usual thickness of the frozen retina and optic nerve sections is 20 and 14 mm, respectively. Finally, eyes were fixed using PFA for wholemount retinas, and retinas were dissected and washed in PBS for subsequent staining.

### Histology and immunostaining

The retinal slices were initially blocked for 2 h in PBS containing 3% bovine serum albumin (BSA), 5% normal goat serum, and 0.4% Triton X-100. Samples were incubated with the primary antibody overnight at 4°C using a blocking solution and then with secondary antibodies for 2 h at room temperature. Whole-mount retinas were then blocked in PBS with 3% BSA and incubated in 5% normal goat serum and 0.6% Triton X-100 for 2 h. Then, primary antibodies were incubated in the blocking buffer for 2 days at 4°C, and secondary antibodies were incubated in the blocking buffer for 2 h at room temperature. Wholemount retinas were blocked in PBS with 3% BSA, 5% normal goat serum, and 0.6% Triton X-100 for 2 h. Next, primary antibodies were incubated for 2 days at 4°C in a blocking buffer, and secondary antibodies were incubated for 2 h at room temperature in a blocking buffer. Before sealing the slices with Antifade Mounting Medium, the slices were washed three times with PBS for 10 min each. Images of immunofluorescence staining were acquired using a spinning disk confocal super-resolution microscope (SpinSR10, Olympus, Japan). The following primary antibodies were used for staining: mouse anti-beta III tubulin (Tuj1, 1:300, Abcam, ab78078), rabbit anti-phospho-S6 ribosomal protein (Ser235/236) (1:200, Cell Signaling Technology, 2211), and guinea pig anti-RNA-binding protein with multiple splicing (anti-RBPMS) (1:200, MilliporeSigma, ABN1376), The following secondary antibodies were used: goat anti-mouse IgG (H + L) Alexa Fluor 488 (1:500, Abcam, ab150113), goat anti-rabbit IgG (H + L) Alexa Fluor 555 (1:500, Abcam, ab150114), and goat anti-guinea pig 488 (1:500, Abcam, ab150185).

### Analysis of RGC survival and pS6^+^ RGC

To quantify the survival rate of RGC, beta III tubulin antibody (Tuj1) was used to immunostain of wholemount retinas according to the steps mentioned above. Six to eight areas from each entire peripheral region of the retina were randomly selected under a 20× objective field of view, and the density of RGCs was calculated per square millimeter. The RGC survival rate in the mouse retina was calculated by dividing the average number of damaged retinal Tuj1 + cells by the average number of undamaged retinas. The magnification of the wholemount retinal immunostaining image was 20×. To analyze the percentage of pS6^+^ RGC, we used RBPMS (a marker for RGC, RNA-binding protein with multiple splicing) to define RGC and pS6 co-immunostaining. Non-adjacent sections in each mouse were selected for analysis, and the percentage of pS6^+^ RGC in each mouse was calculated by dividing the number of pS6^+^/RBPMS^+^ cells by the number of RBPMS^+^ cells. Only the cells in the ganglion cell layer were counted.

### Quantification of axonal regeneration

Quantification of axons was performed according to previously described approach (Park et al., 2008). The number of regenerated axons was determined by counting the number of CTB-labeled axons at different distances from the crush site in four sections of each nerve. To determine the number of axons per millimeter of nerve width, the cross-sectional width of the nerve was measured at 200, 400, 600, 800, and 1,000 mm distal to the site of the lesion. Then, the average number of axons per millimeter across all sections was calculated. Σad, the total number of axons in a nerve with a radius of r that extends a distance of d, was calculated by adding up all of the sections that were a specific thickness t (14 μm): Σad = πr^2^ × [average axons/mm]/t.

### Statistical analyses

Statistical analysis was done with GraphPad Prism 8, and the significance level was set at *p* < 0.05. Unless specifically indicated, data are presented as mean ± SEM in the graphs. The two-tailed unpaired or paired *t*-test was used to compare the two groups. Comparisons between two groups at multiple time points were analyzed via two-way analysis of variance (ANOVA) by Bonferroni’s *post-hoc* test.

## Results

### Ocular administration of HupA promotes RGC survival after ONC

Previous reports found a neuroprotective effect of HupA on RGCs (Yu et al., 2021). To further investigate the protective role of HupA on RGCs after ONC, we used intraperitoneal injection, ocular drop, and intravitreal injection. Intraperitoneal injection and ocular drop of HupA were administered daily for 14 days, while intravitreal injection of HupA was administered six times (Figure 1A). To detect the survival of RGC in the retina, we performed an immunostaining analysis of the whole retina with beta III tubulin (Tuj1) antibody (Figure 1B). In the intraperitoneal treatment experiment, the RGC density of the 0.2% HupA group was 526 ± 48 Tuj1^+^ cells/mm^2^, and that of the control group was 547 ± 31 Tuj1^+^ cells/mm^2^ (Figures 1C, F). These results showed that intraperitoneal injection of HupA did not promote the survival of RGCs after ONC. In the eye drop treatment experiment, the RGC density treated with HupA at concentrations of 0.05, 0.1, and 0.2% were 530 ± 22 Tuj1^+^ cells/mm^2^, 533 ± 37 Tuj1^+^ cells/mm^2^, and 585 ± 52 Tuj1^+^ cells/mm^2^, respectively. The ocular administration experiment showed that the density of surviving RGCs in the 0.2% HupA group was slightly higher than that in the control group (*p* = 0.0173) (Figures 1D, G). In the intravitreal treatment experiments, the density of surviving RGCs in the HupA-treated groups with concentrations of 0.05, 0.1, and 0.2% were 577 ± 51 Tuj1^+^ cells/mm^2^, 664 ± 58 Tuj1^+^ cells/mm^2^, and 1,030 ± 142 Tuj1^+^ cells/mm^2^, respectively. Obviously, the results of intravitreal injection group show a significant increase in the survival of RGCs in the 0.2% HupA treatment group compared to the control group (*p* < 0.0001) (Figures 1E, H).

In addition, we investigated the effect of different doses of HupA on the survival rate of RGC. The above results showed that both ocular drops and intravitreal injections of HupA promote the survival of RGCs after ONC. Although the density of RGCs was elevated in the ocular drip 0.2% HupA group, its protective effect was limited. In contrast, the density of surviving RGCs was significantly elevated by intravitreal injection administration.

### Intravitreal injection of HupA promotes RGC axonal regeneration after optic nerve crush

**FIGURE 2:**
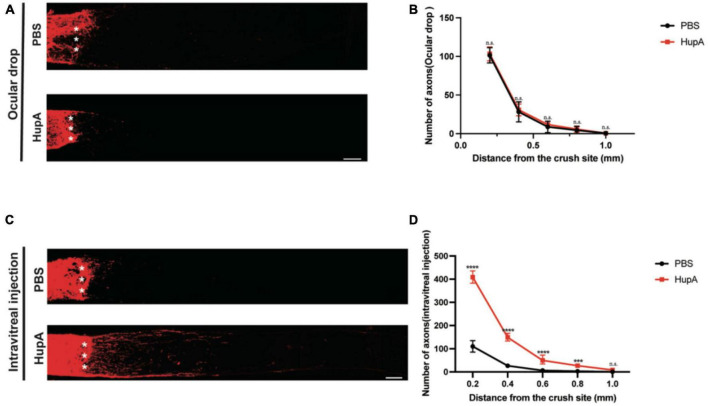
Intravitreal injection of Hup-A promotes RGCs axonal regeneration after ONC. **(A)** Confocal images of CTB-555 tracing of RGC axons in the optic nerve from ONC 2w eyes treated with ocular drops of PBS and HupA. The asterisk represents the site of optic nerve crush: scale bar, 100 mm. **(B)** Quantification of the axonal regeneration in **(A)** (two-way ANOVA followed by Bonferroni’s multiple-comparisons test, *n* = 5, *p > 0.05). **(C)** Confocal images of anterograde CTB tracing of RGC axons in the optic nerve from ONC 2w eyes treated with intravitreal injection of PBS and HupA. The asterisk represents the site of optic nerve crush: Scale bar, 100 μm. **(D)** Quantification of the axonal regeneration in **(C)** (Data are represented as mean ± SEM, two-way ANOVA followed by Bonferroni’s multiple-comparisons test, *n* = 5 in each group, ****p < 0.0001 at 0.2, 0.4, and 0.6 mm, ***p < 0.001 at 0.8 mm from the crush site).

Several natural products have a protective effect on RGCs (Sim et al., 2022). However, only a few natural products can facilitate the RGC axon regeneration after ONC. In this study, we found that ocular administration could promote the survival of RGCs after ONC. Next, we investigated whether ocular drop and intravitreal injection of HupA promote RGC axonal regeneration after ONC. Intravitreal injection of CTB-555 was used to track the regenerating axons of the RGCs at the 12th day post crush (12dpc). When using ocular drop treatment, no significant axonal regeneration in either the HupA treatment group or the control group was observed (Figures 2A, B). The intravitreal injection treatment experiment showed minimal optic nerve regeneration in the control group. However, optic nerve regeneration was significantly improved by intravitreal injection of HupA (Figure 2C). It should be noted that most of the regenerating axons of the optic nerve reach 500 mm from the site of crush, with the longest regenerating axons being over 1,000 mm from the site of crush (Figure 2D). These results suggested that intravitreal injection of HupA promotes the RGC axonal regeneration after ONC.

### Intravitreal injection of HupA can enhance mTOR activity after ONC

**FIGURE 3:**
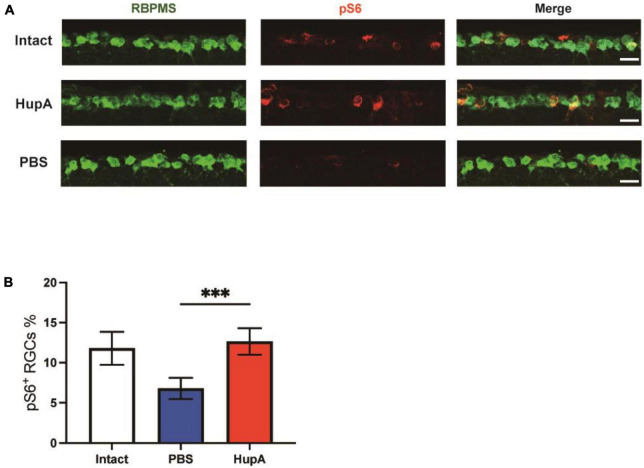
Intravitreal injection of HupA can enhance mTOR activity after ONC. **(A)** Representative retinal sections demonstrate that HupA increases mTOR activity (marked by pS6, red) in the RGC (RBPMS, green). The intact group has no optic nerve damage. Both the HupA and PBS groups were 3 days post optic nerve injury. Scale bar, 20 μm. **(B)** Quantification of the percentage of ps6^+^ RGCs in **(A)** (Data are represented as mean ± SEM, unpaired *t*-test, ^***^p < 0.001, *n* = 5 in each group).

To investigate whether HupA affects mTOR activity in RGC after ONC, we evaluated mTOR activity by immunostaining the phosphorylation levels of ribosome protein S6 (a marker for mTOR activation). In immunostaining of retinal sections, RBPMS (RGC marker, RBPMS, RNA-binding protein with multiple splicing) antibody was used to identify RGC. In undamaged retinas, the percentage of pS6-positive (pS6^+^) RGCs was 11.8 ± 1.9%. HupA treatment significantly improved the percentage of pS6^+^ RGCs compared to the control group at 3 dpc (*p* = 0.003) (Figures 3A, B). According to quantitative findings, the percentage of ps6^+^ RGCs in the HupA-treated group was 12.7 ± 1.5% at 3 dpc, while it was 6.8 ± 1.2% in the control group. These results indicated that intravitreal injection of HupA could maintain mTOR activity after ONC.

### The effect of HupA on promoting axonal regeneration can be rescued by rapamycin

**FIGURE 4:**
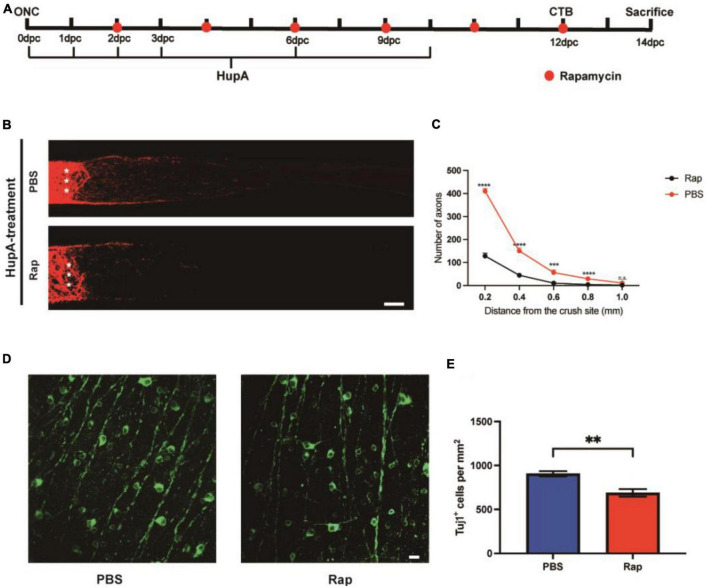
The effect of HupA on promoting axonal regeneration can be rescued by rapamycin. **(A)** Experimental timeline. PBS or rapamycin (Rap) was given intraperitoneally once every 2 days with HupA-treatment (Crush, optic nerve crush; CTB, intravitreal injection of cholera toxin β-subunit; dpc, days post-crush). **(B)** Confocal images of CTB-555 tracing of RGC axons from the mice with HupA-treatment after ONC and intraperitoneal injection of PBS or rapamycin (The asterisk represents the site of optic nerve crush. Scale bar, 100 mm). **(C)** Quantification of the axonal regeneration in (A) (two-way ANOVA followed by Bonferroni’s multiple-comparisons test, *n* = 5 mice in each group, ****p < 0.0001 at 0.2, 0.4 mm from the crush site; ***p < 0.001 at 0.6 mm from the crush site, *p < 0.05 at 0.8 mm from the crush site). **(D)** Representative confocal images of wholemount retinas showing RGCs survival (Tuj1^+^) in the rapamycin and control groups. Scale bar, 20 μm. **(E)** RGC density (Tuj1^+^ cells/mm^2^) was measured from **(D)** (Data are represented as mean ± SEM, unpaired *t*-test, ^**^p < 0.01, *n* = 6 in each group).

To examine whether HupA could promote RGC survival and axonal regeneration through mTOR, we used a specific inhibitor of mTOR, rapamycin (Guertin and Sabatini, 2007). The mice were divided into two groups after HupA treatment, and rapamycin or sterile saline was administered intraperitoneally every 2 days (Figure 4A). We observed a significantly reduced quantity and length of axons in the rapamycin-injected group compared to the PBS-injected group (Figure 4B). After rapamycin injection, most of the regenerating axons were restricted to 300 μm from the site of crush, and longest axons did not exceed 600 mm (Figure 4C). In addition, the density of surviving RGCs in the intraperitoneal injection of rapamycin group and the control group was 689 ± 101 Tuj1^+^ cells/mm^2^ and 906 ± 49 Tuj1^+^ cells/mm^2^, respectively. The above results indicate that compared to the control group, the survival rate of RGCs was reduced by rapamycin (*p* = 0.002) (Figures 4D, E). Taken together, the results showed that rapamycin could reduce the neuroprotective and axonal regenerative effects of HupA after ONC.

## Discussion

The RGCs located in the retina and axons converge to form the optic nerve, and have an essential role in transmitting information from the retina to the brain (Zhang et al., 2022). Due to their unique anatomy, RGCs are susceptible to injury, including traumatic injury, ischemia/reperfusion (I/R) injury, and chronic inflammation (Fague et al., 2021). Yet, similar to the other central nervous system (CNS) components, RGCs have almost no intrinsic ability for axonal regeneration after injury (Gokoffski et al., 2020). Therefore, research on optic nerve protection has focused on maintaining RGC after injury and encouraging optic nerve axon regeneration (Laha et al., 2017).

In this study, we investigated the ability of a natural extract to the neuroprotection of RGCs using a mouse ONC model. Interestingly, we found that this natural extract affects RGC axon regeneration. Natural extracts are receiving increasing attention in promoting neuronal survival and neurogenesis after neuronal damage and neurodegeneration (Zhao et al., 2015; Ige and Liu, 2020). With fewer side effects and low cost, natural extracts have become one of the most commonly used complementary and alternative medicine therapies in treating neurodegenerative diseases (Sharifi-Rad et al., 2020). However, natural extracts are rarely used to treat neurotraumatic disorders, especially in TON.

Although several treatment options are available for TON, including surgery and medication, doctors have not yet reached a consensus on the best treatment method (Lai et al., 2015). A corticosteroid regimen, surgical decompression, or a combination of the two can be used to reduce secondary inflammation and edema (Li et al., 2021). However, the surgical treatment has unsatisfactory results and can lead to postoperative infections and other iatrogenic complications (Chaon and Lee, 2015). On the other hand, corticosteroids are common drugs in pharmacotherapy, yet, high-dose corticosteroids may lead to mild to life-threatening complications, including glaucoma, cataracts, and osteoporosis (Li et al., 2021).

Compared to corticosteroids, several natural products, such as saffron, *G. biloba*, and marijuana, have fewer side effects and can lower intraocular pressure (Sim et al., 2022). In addition, a handful of natural herbal products have neuroprotective effects and potential therapeutic applications in neurotrauma (More et al., 2012). For example, the main catechin in green tea, epigallocatechin-3-gallate, can promote RGC survival following ONC when administered intraperitoneally and orally (Xie et al., 2010). Moreover, intravitreal injection of the citrus component naringenin increases the RGC survival rate after ONC (Chen et al., 2021). However, so far, only a few reports have investigated the effect of natural herbal products on neuronal regenerative capacity.

Huperzine A (HupA) is a natural product extract that can easily cross the blood-brain barrier with no signs of cytotoxicity and few side effects (Xu et al., 2014). HupA has been reported as an acetylcholinesterase (AChE) inhibitor and excessive AChE activity is known to cause damage to the CNS (Xu et al., 2014; Damar et al., 2016). Therefore, we also examined changes in AChE activity before and after optic nerve injury and after HupA treatment. The results showed that HupA was effective in reducing the activity of AChE in retinal tissue (Supplementary Figure 3). Yet, to date, only one study has reported the potential retinal protective ability of HupA in a retinal ischemia/reperfusion (I/R) injury and oxygen-glucose deprivation (OGD)-induced neuronal damage model, which found that HupA can protect RGCs (Yu et al., 2021). Compared with the OGD-induced model, the pathogenesis of the ONC model, which was used in this study, is different. Although retinal I/R and OGD-induced neuronal damage can cause varying degrees of RGC injury, they are unsuitable for studying axonal regeneration due to the variable nature of insult and incomplete degeneration of RGC axons (Cameron et al., 2020).

Huperzine A (HupA) has been reported to decrease the inflammatory response and reduce neuronal apoptosis after spinal cord injury (Antar et al., 2018). However, these studies only confirmed that HupA could reduce the apoptosis of neurons after damage, and it remains unclear whether HupA affects the regenerative capacity of neurons after injury. Furthermore, the survival of neurons does not mean they can regain their original function (He and Jin, 2016). Hence, it is also necessary to enhance their regenerative capacity (Tran et al., 2018). Unfortunately, natural product studies have rarely used axonal tracing techniques to observe axonal regeneration, although it is the crucial aspect of functional recovery.

In this study, we investigated the effect of HupA on RGC survival and axonal regeneration following ONC, with immunofluorescence of wholemount retina and intravitreal injection of CTB-555. More importantly, we used three administration methods to better assess the neuroprotective and regenerative capacity of HupA. After ONC, mice treated with 0.2% HupA eye drops showed a surviving RGC density of approximately 585 Tuj1^+^ cells/mm^2^ after 2 weeks. In contrast, the density of surviving RGCs (Tuj1^+^ cells/mm^2^) was approximately 1,030 in mice treated with intravitreal injection of 0.2% HupA, which was significantly higher than that observed with eye drops. Ocular drops are common and widely used approach for drug delivery; yet, this method has very low bioavailability (Maulvi et al., 2016). Compared with an ocular drop, the frequency and dosing of intravitreal injection were much lower in this study. Meanwhile, we also found that intravitreal injection could significantly promote axonal regeneration after ONC. Interestingly, the axonal regeneration was not obvious when ocular drops were applied.

Intravitreal injection has been used as a common method of drug delivery in ophthalmology (Lai et al., 2015). Intravitreal injections of anti-vascular endothelial growth factor (VEGF) are commonly used to treat retinal diseases, as they allow the drug to act directly on the neovascular lesion and obtain better bioavailability through this delivery method (de Cogan et al., 2017). For axonal regeneration, we believe that intravitreal injection can achieve better bioavailability and higher RGC survival than other delivery methods (Maulvi et al., 2021). However, frequent intravitreal injections might lead to an inflammatory response, limiting its clinical applicability (Lai et al., 2015). Therefore, future studies are needed to further explore reducing the frequency of drug delivery and optimizing the delivery methods.

Furthermore, we also revealed the molecular mechanism through which HupA promotes RGC survival and axonal regeneration following ONC. Park et al. demonstrated that activation of phosphatidylinositol 3 kinase (PI3K) by deleting phosphatase and tensin homologs (PTEN) induces axonal regeneration in the CNS by activating mammalian target of rapamycin (mTOR) signaling (Park et al., 2008). Phosphorylation of ribosomal protein S6 (pS6) is often used as a marker of mTOR activation, and antibodies to pS6 have been used in several studies to track the mTOR activity in RGCs (Chen et al., 2020; Wang et al., 2020). Previously, HupA was found to protect HT22 cells from glutamate neurotoxicity by regulating the PI3K/AKT/mTOR pathway, in which phosphorylation of AKT and mTOR was increased in the HupA-treated group (Mao et al., 2016). HupA also preserved primary retinal neurons from OGD-induced injury by an indirect activation-mediated protein kinase B/mitogen-activated protein kinases (AKT/MAPK) pathway (Yu et al., 2021). The above reports have only investigated changes in AKT/mTOR signaling pathways in other cells or the total retina; yet, there is no accurate observation of changes in mTOR activity in the RGC. To investigate whether HupA can affect mTOR activity in RGC after ONC, we examined the changes of pS6 3 days after ONC. We found that the percentage of pS6^+^ RGCs in the HupA-treatment group was approximately twice that of pS6^+^ RGCs in the control group, indicating that HupA can enhance the activity of mTOR. In addition, we used rapamycin to rescue the effect of HupA on RGCs and found that rapamycin blocked most of the therapeutic effects. However, a little residual axonal regeneration was observed in the rapamycin group, suggesting that other factors might be involved in the axonal regeneration and neuroprotective effects of HupA.

Although many scholars have reported that intraperitoneal injection of rapamycin in CNS research can block neuroprotective and axon regeneration effects through the mTOR pathway, several studies have shown that rapamycin may improve the survival rate of RGCs (Li et al., 2016). Rodríguez-Muela et al. (2012) found that intraperitoneal injection of rapamycin can inhibit mTOR activation, which can help promote RGC survival and axon regeneration by removing damaged or dysfunctional cellular components and enhancing cellular metabolism. Wen et al. (2019) used the ONC model to illustrate that rapamycin’s inhibition of RGC survival may be achieved by inhibiting mTORC1 rather than mTORC2. Based on these reports, rapamycin may have opposite effects on RGC protection in different studies. We have also presented relevant experiments in the Supplementary material, which show that the use of rapamycin alone does not seem to have an effect on the survival of RGCs after optic nerve injury. Overall, the effects of rapamycin on RGC survival after optic nerve injury are complex and may depend on multiple factors. Further research is needed to fully understand the mechanisms underlying these effect.

There are some limitations to this study. Although we observed that vitreous cavity injection of HupA can promote RGC survival and axon regeneration, we have not observed whether its visual function has improved. In addition, we have not determined whether HupA influences other factors in the retina that exert neuroregenerative effects. These questions will need to be determined in future studies.

In summary, we found that the herbal extract HupA could alter the mTOR pathway in RGCs, thereby promoting optic nerve regeneration. These results strongly support that HupA is a potential therapeutic strategy for TON.

## Data availability statement

The original contributions presented in this study are included in the article/Supplementary material, further inquiries can be directed to the corresponding authors.

## Ethics statement

The animal study was reviewed and approved by the Institutional Animal Care and Use Committee of Shanghai Sixth People’s Hospital Affiliated to Shanghai Jiao Tong University, Shanghai, China. Written informed consent was obtained from the owners for the participation of their animals in this study.

## Author contributions

L-YZ, DC, D-FF, and T-CL contributed to the conception or design of the work and drafted the manuscript. L-YZ and X-RG analyzed the data. Y-QN and Y-SX contributed to the supplementary experiments. All authors reviewed and approved the final manuscript.

## Supplementary material

Supplementary Figure 1 Immunofluorescence staining of retinal sections at 14 days after ONC. **(A)** Representative confocal images of retinal sections showing surviving RBPMS RGCs using the ocular drip drug delivery method in the HupA-treatment and control mouse groups. Scale bar, 20 μm. **(B)** Quantification of the RGC survival rate in **(A)** (unpaired *t*-test, *****p* < 0.0001; *n* = 5 mice in each group, at least eight non-adjacent retinal sections per mouse). **(C)** Representative confocal images of retinal sections showing surviving RBPMS RGCs using vitreous cavity injection administration in the HupA-treatment and control mouse groups. Scale bar, 20 μm. **(D)** Quantification of the RGC survival rate in **(C)** (Data are represented as mean ± SEM, unpaired *t*-test, *****p* < 0.0001; *n* = 5 mice in each group, at least eight non-adjacent retinal sections per mouse).

Supplementary Figure 2 Intravitreal injection of HupA increases total retinal mTOR activity after optic nerve injury. **(A)** Western blot analysis of S6 phosphorylation levels in retinas treated with HupA on day three after optic nerve injury. **(B)** Quantification of the Western blot in **(A)** (Data represent mean ± SEM, unpaired *t*-test, ****p* < 0.001, *n* = 3 in each group).

Supplementary Figure 3 HupA injection in the vitreous cavity effectively reduces the activity of AchE in the retina. Quantitative analysis of retinal AchE activity in intact mice was 0.54 ± 0.13. Quantitative analysis of retinal AchE activity in mice after optic nerve injury was 0.89 ± 0.13. Quantitative analysis of retinal AchE activity in mice injected with HupA intravitreally for 3 days after optic nerve injury was 0.57 ± 0.1 (Data are represented as mean ± SEM, unpaired *t*-test, **p* < 0.05, *n* = 4 in each group). The cholinesterase activity was assayed with a commercial kit (Acetylcholinesterase Activity Assay Kit, Solarbio) according to the manufacturer’s instructions.

Supplementary Figure 4 Intraperitoneal injection of rapamycin can effectively inhibit the activity of mTOR in RGC. **(A)** Representative retinal sections demonstrate that rapamycin decreases mTOR activity (marked by pS6, red) in the RGC (RBPMS, green). Scale bar, 20 μm. **(B)** Quantification of the percentage of pS6^+^ RGCs in **(A)** (Data are represented as mean ± SEM, unpaired *t*-test, *****p* < 0.0001, *n* = 5 in each group).

Supplementary Figure 5 Intraperitoneal injection of rapamycin is not effective in promoting RGC survival and axonal regeneration after ONC. **(A)** Confocal images of CTB-555 tracing of RGC axons in the optic nerve from ONC 2w eyes treated with intraperitoneal injection of PBS and rapamycin. The asterisk represents the site of optic nerve crush. Scale bar, 50 mm. **(B)** Quantification of the axonal regeneration in **(A)** (Data are represented as mean ± SEM, two-way ANOVA followed by Bonferroni’s multiple-comparisons test, *n* = 5 in each group).

Supplementary Figure 6 Intravitreal HupA injection has a better neuroprotective effect. Comparison of RGC survival rates between eye drop application and intravitreal injection. In the ocular drop treatment experiment, the density of surviving RGCs after treatment with 0.2% concentration of HupA was approximately 585 Tuj1^+^ cells/mm^2^. In the intravitreal treatment experiments, the density of surviving RGCs after treatment with 0.2% concentration of HupA was approximately 1,030 Tuj1^+^ cells/mm^2^ (Data are represented as mean ± SEM, unpaired *t*-test, *****p* < 0.0001; *n* = 6 mice in each group).

Supplementary Figure 7 The survival of RGCs after ONC with rapamycin treatment alone. **(A)** Experimental timeline: mice will be randomly divided into two groups and treated with rapamycin at different doses and frequencies (Crush: optic nerve crush). **(B)** The confocal images of retinal flat-mount stained with Tuj1^+^ immunofluorescence show the surviving RGCs at 2 weeks after ONC in three different groups: control (PBS), 6 mg/kg rapamycin, or 7.5 mg/kg rapamycin. Scale bar, 20 μm. **(C)** Quantification of surviving RGCs density (Tuj1^+^ cells/mm^2^) in each treatment group 2 weeks after ONC, measured from images such as those in **(B)** (Data are represented as mean ± SEM, unpaired *t*-test, *n* = 5 mice in each group).
