# Supplementary figures and images for: Intravitreal injection of Huperzine A promotes retinal ganglion cells survival and axonal regeneration after optic nerve crush
Source: Front Cell Neurosci. 2023 May 24;17:1145574. doi: 10.3389/fncel.2023.1145574 (PMC10244636; doi:10.3389/fncel.2023.1145574)

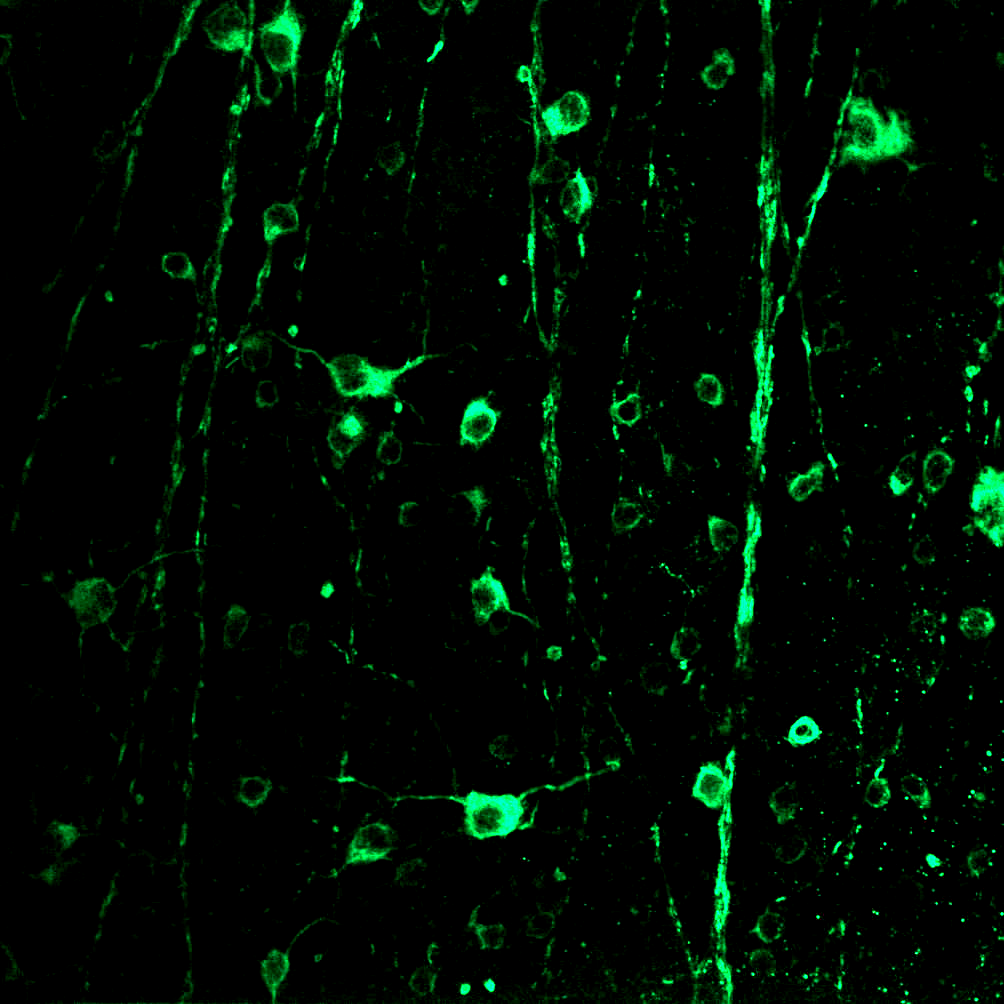

Supplement: Supplementary file 1 [file Data_Sheet_1.ZIP › Raw date/Figure 4/Density of RGCs at ONC 14d/Rap.tif]

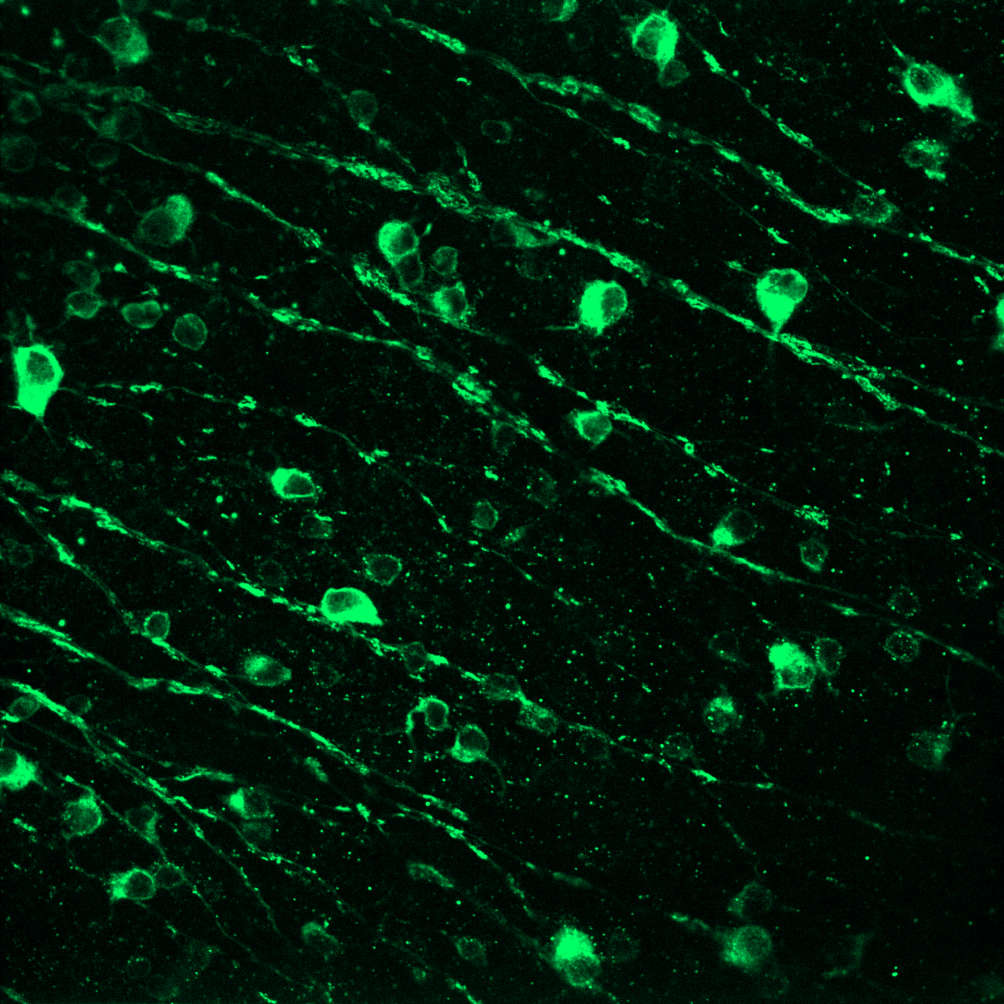

Supplement: Supplementary file 1 [file Data_Sheet_1.ZIP › Raw date/Figure 4/Density of RGCs at ONC 14d/PBS.png]

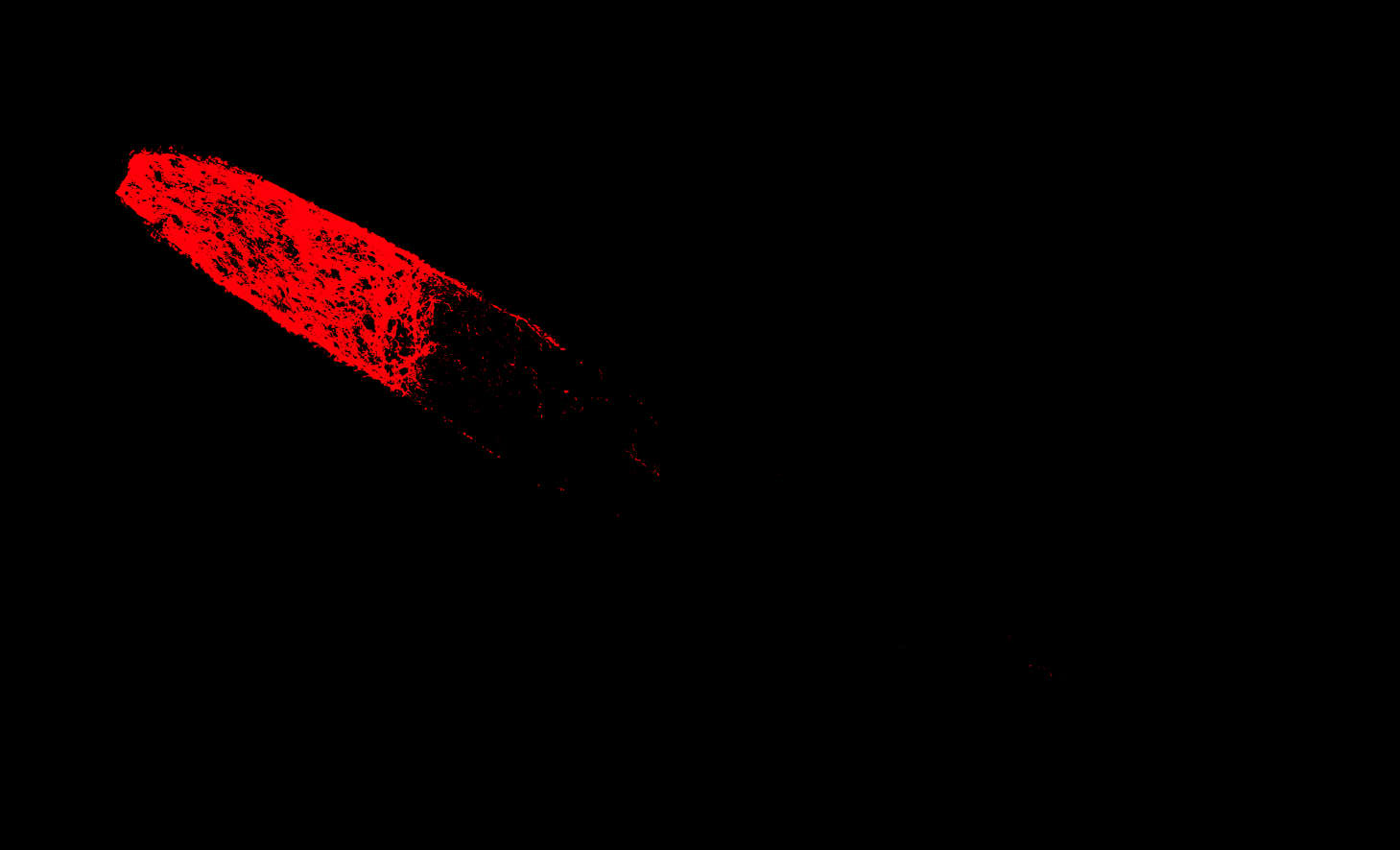

Supplement: Supplementary file 1 [file Data_Sheet_1.ZIP › Raw date/Figure 4/Axon/Rap.png]

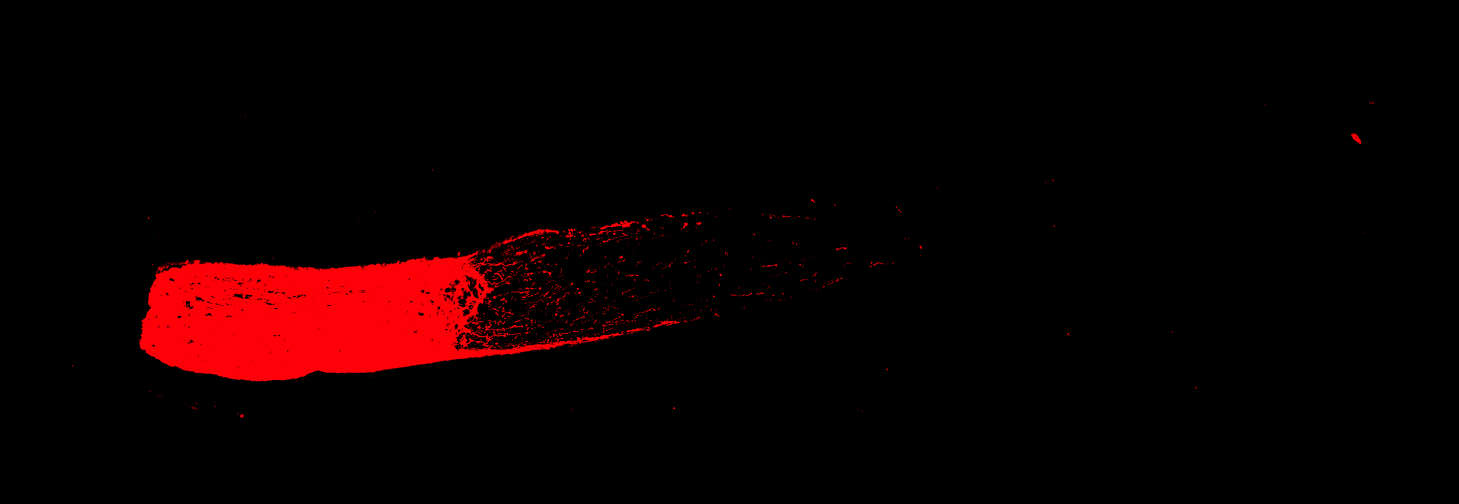

Supplement: Supplementary file 1 [file Data_Sheet_1.ZIP › Raw date/Figure 4/Axon/PBS.png]

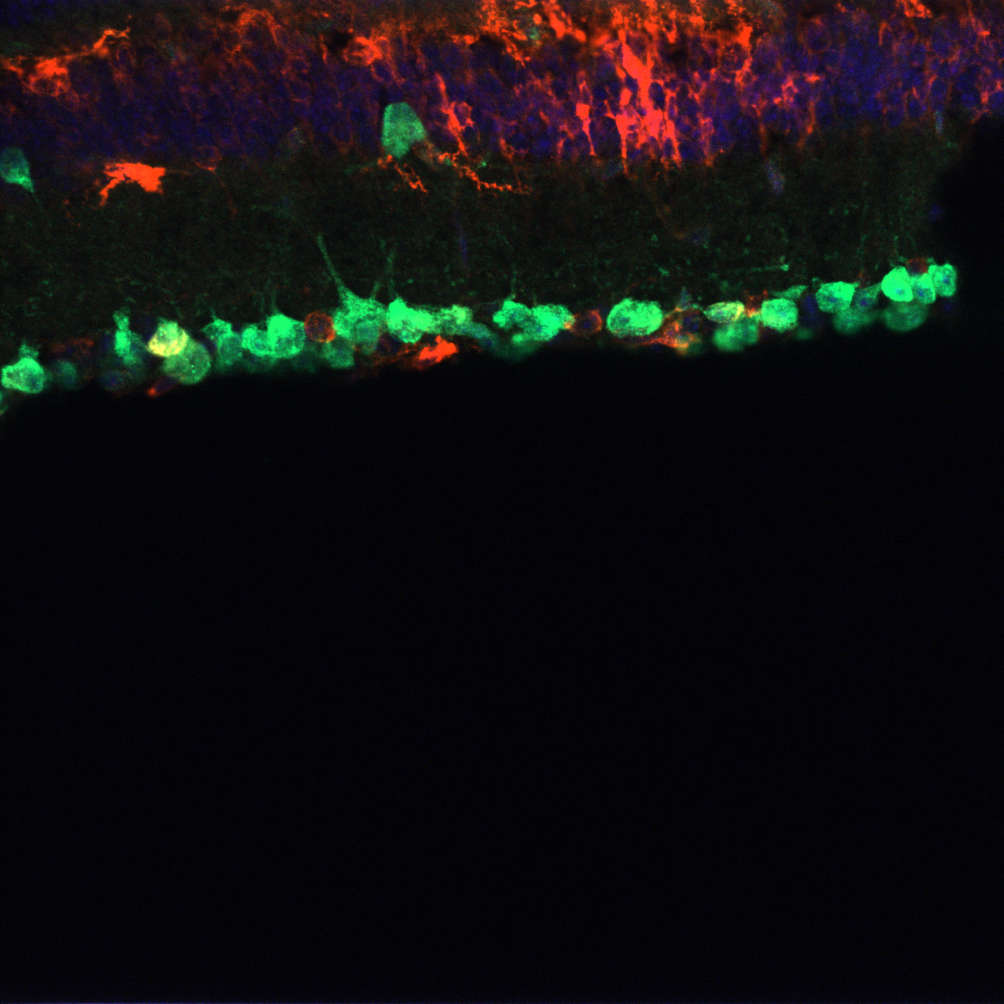

Supplement: Supplementary file 1 [file Data_Sheet_1.ZIP › Raw date/Figure 3/Original picture/intact.png]

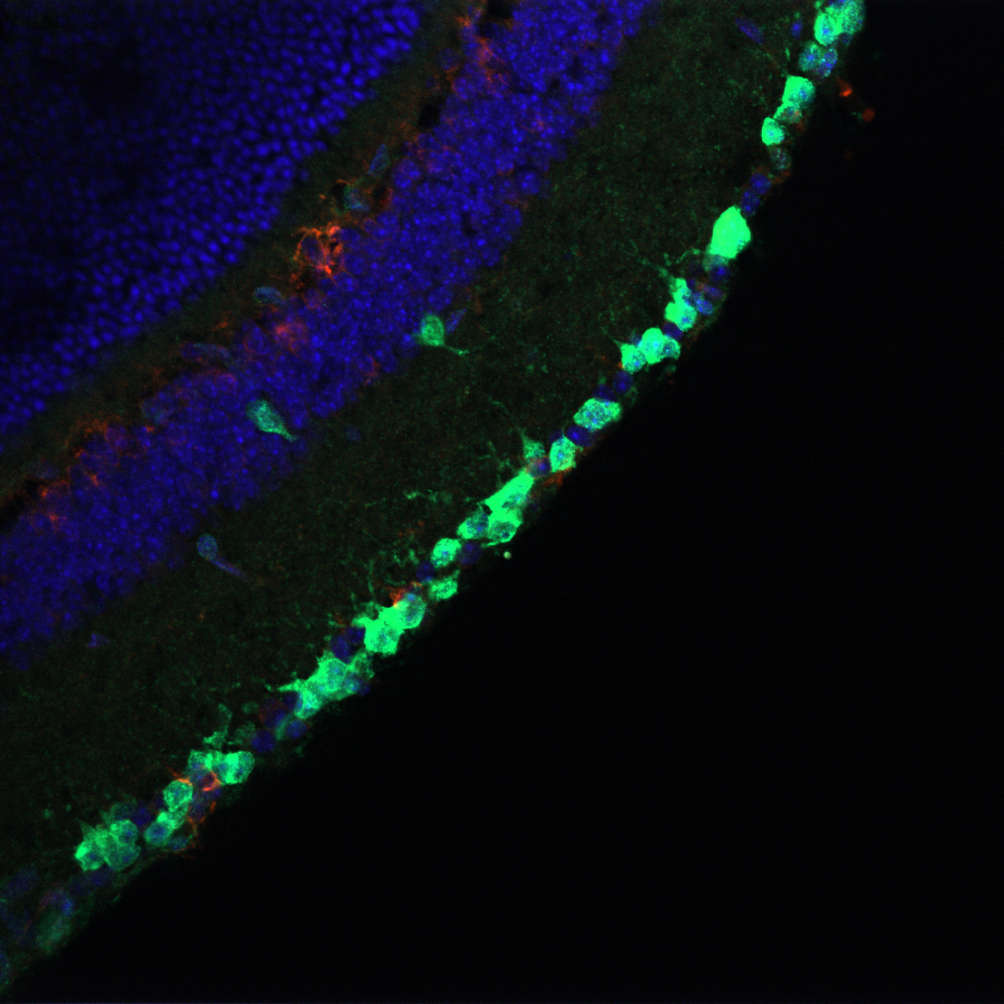

Supplement: Supplementary file 1 [file Data_Sheet_1.ZIP › Raw date/Figure 3/Original picture/PBS.png]

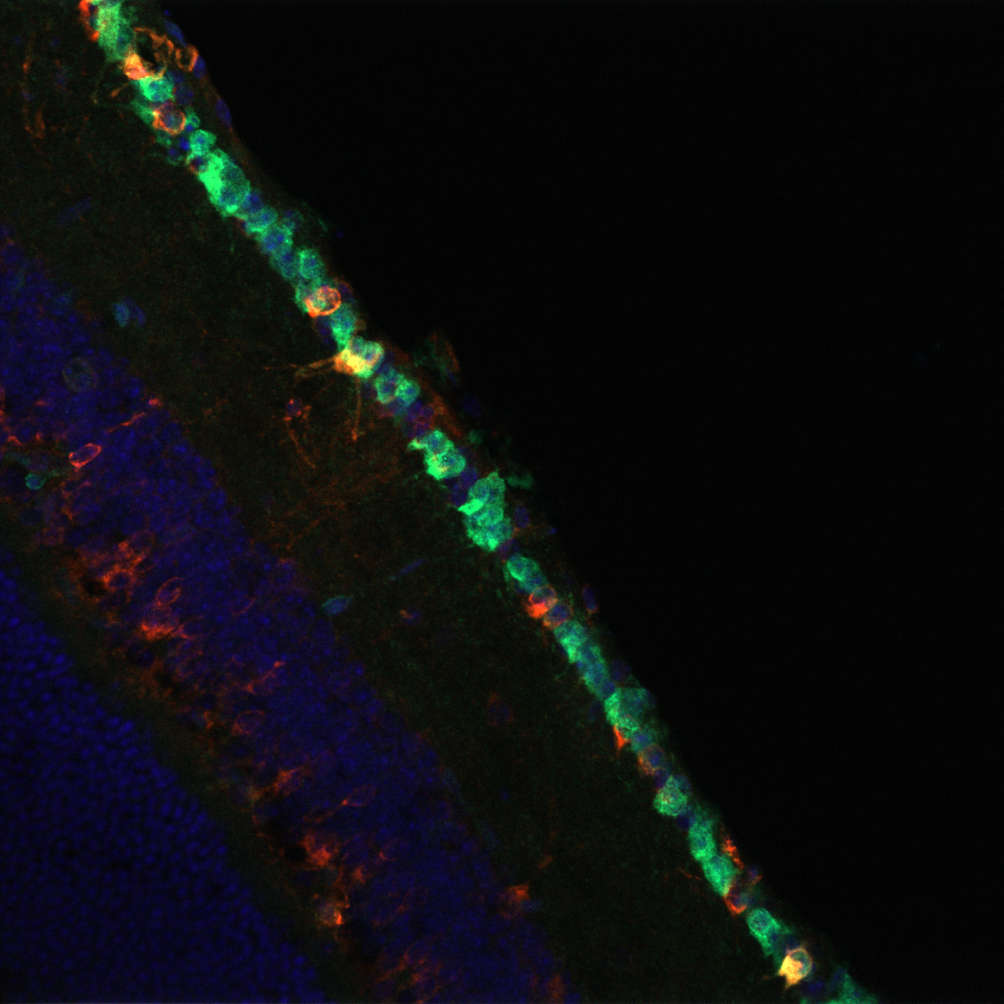

Supplement: Supplementary file 1 [file Data_Sheet_1.ZIP › Raw date/Figure 3/Original picture/HupA.png]

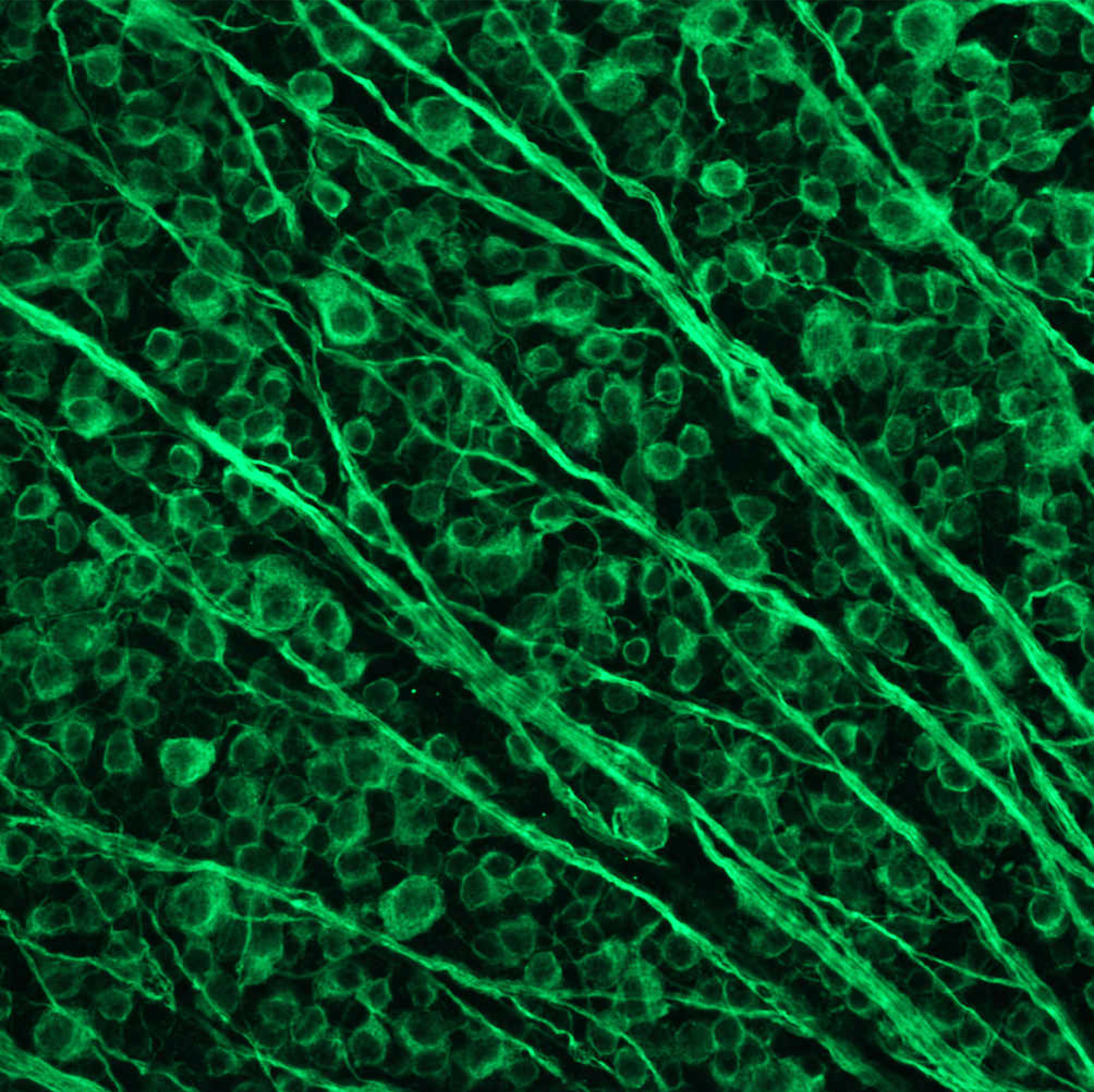

Supplement: Supplementary file 1 [file Data_Sheet_1.ZIP › Raw date/Figure 1/Original picture/Intact /Intact.jpg]

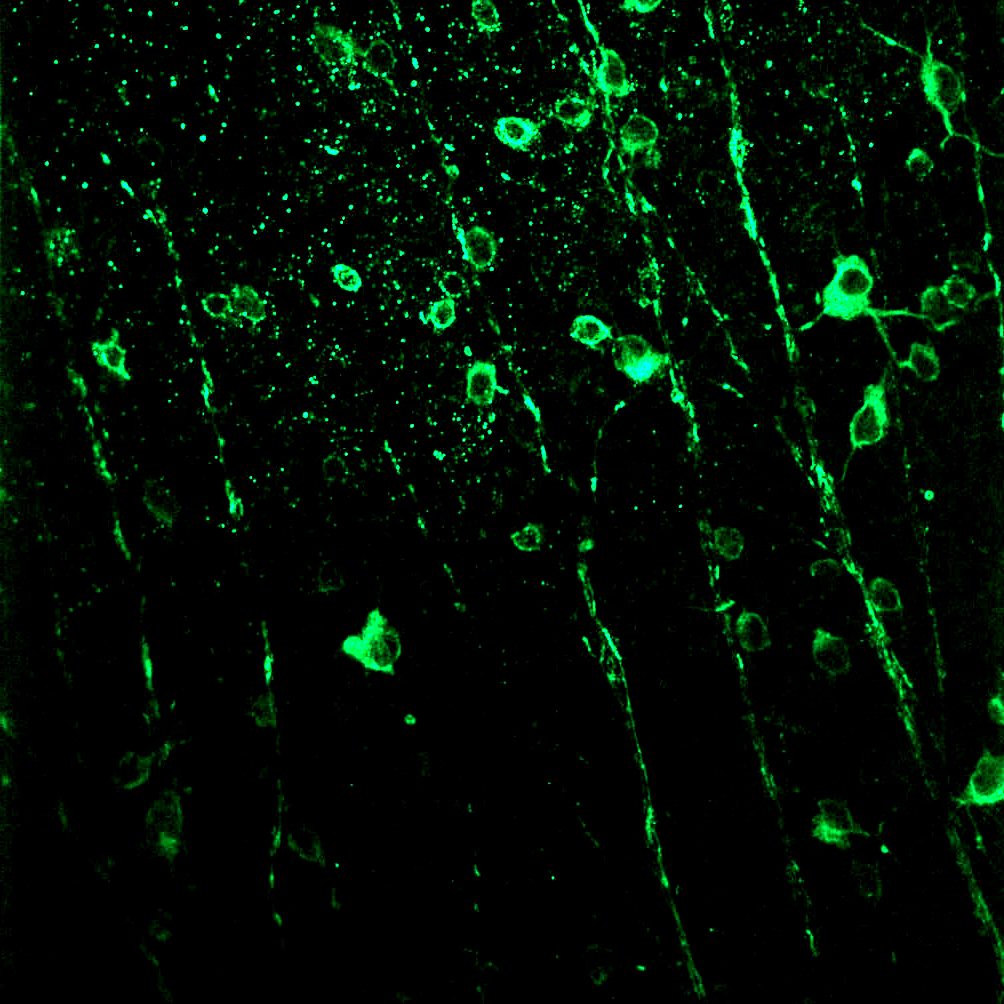

Supplement: Supplementary file 1 [file Data_Sheet_1.ZIP › Raw date/Figure 1/Original picture/eye drop/Drop 0.05%HupA_.jpg]

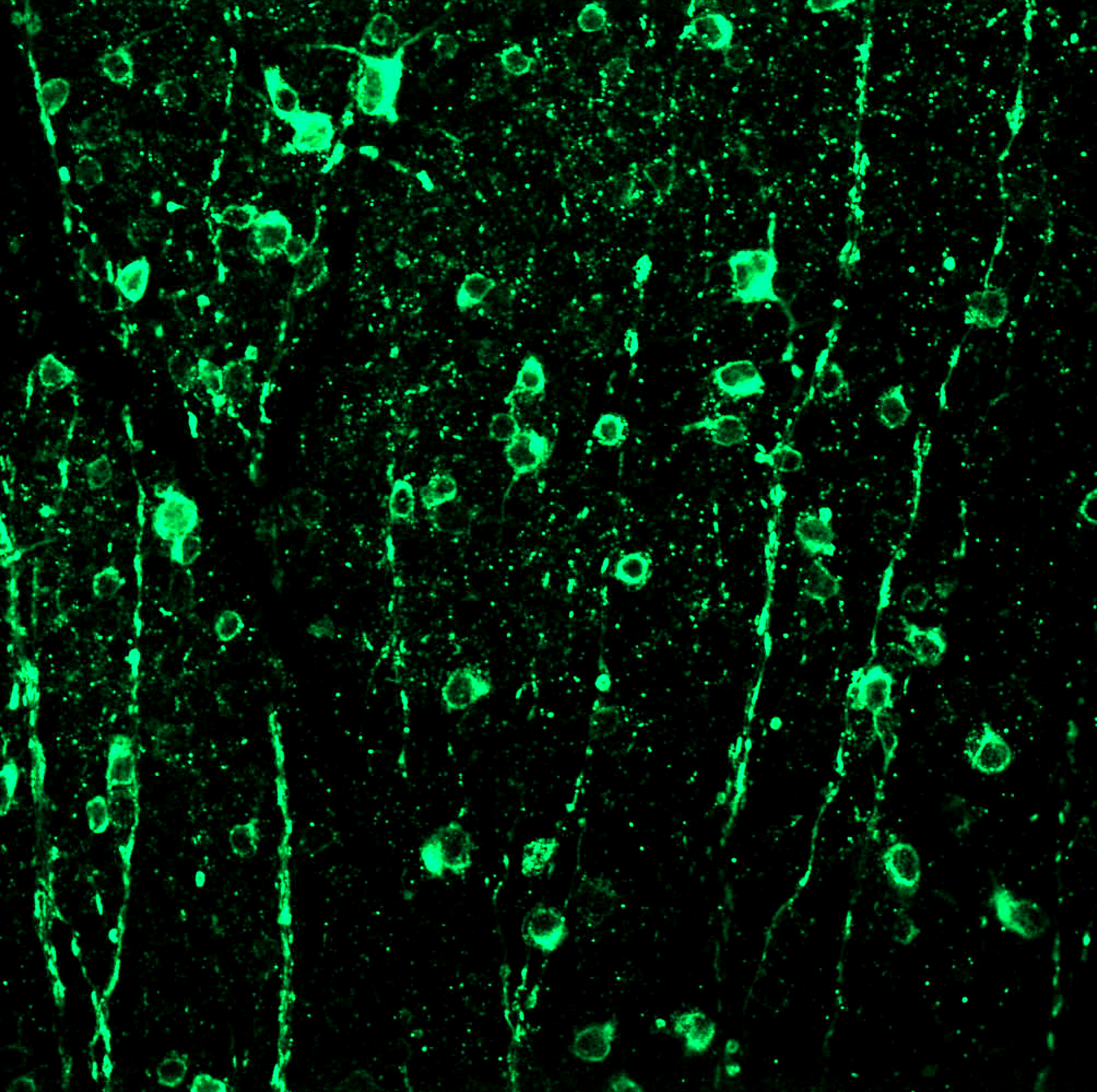

Supplement: Supplementary file 1 [file Data_Sheet_1.ZIP › Raw date/Figure 1/Original picture/eye drop/Drop 0.2%HupA.jpg]

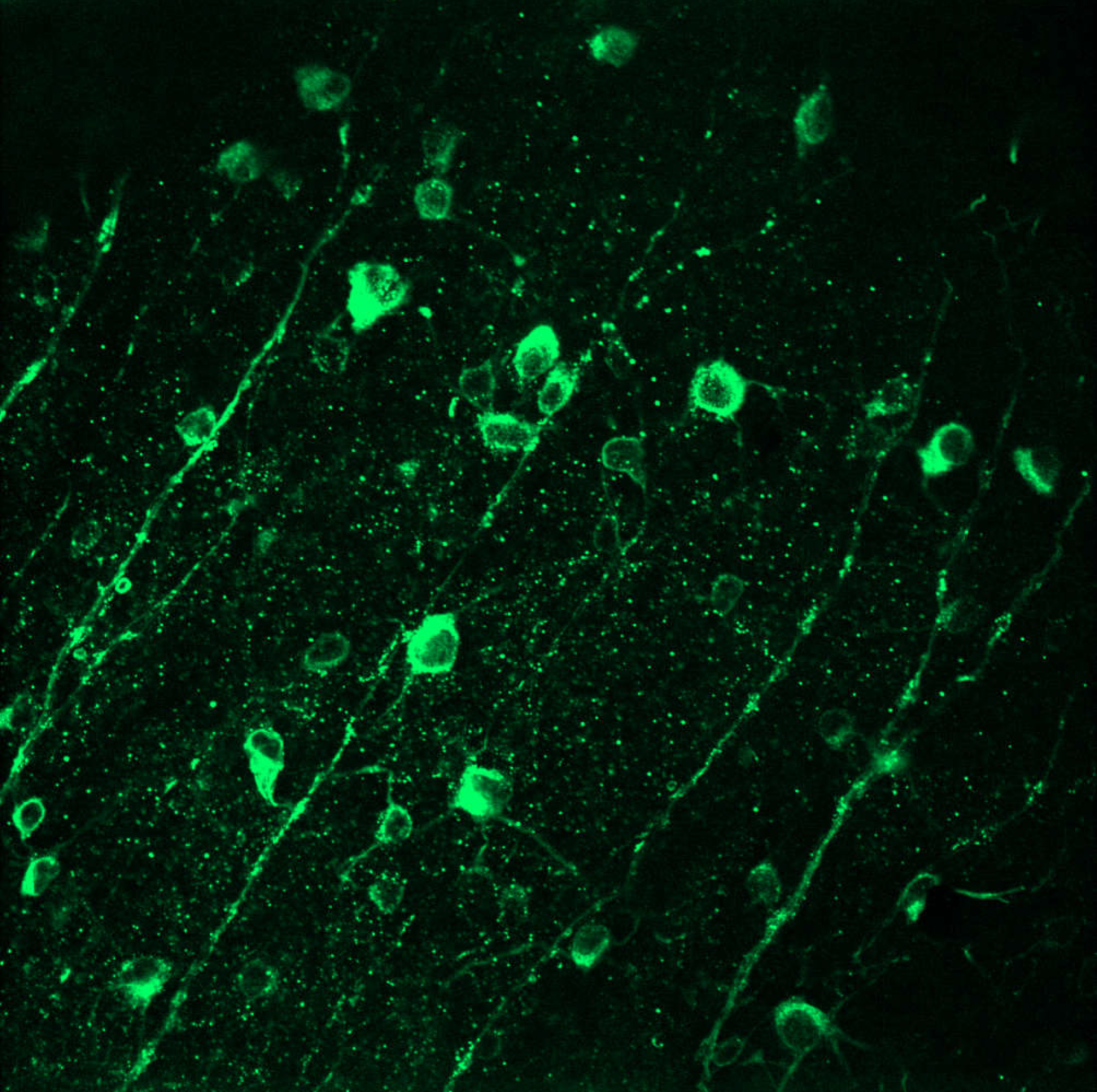

Supplement: Supplementary file 1 [file Data_Sheet_1.ZIP › Raw date/Figure 1/Original picture/eye drop/PBS.jpg]

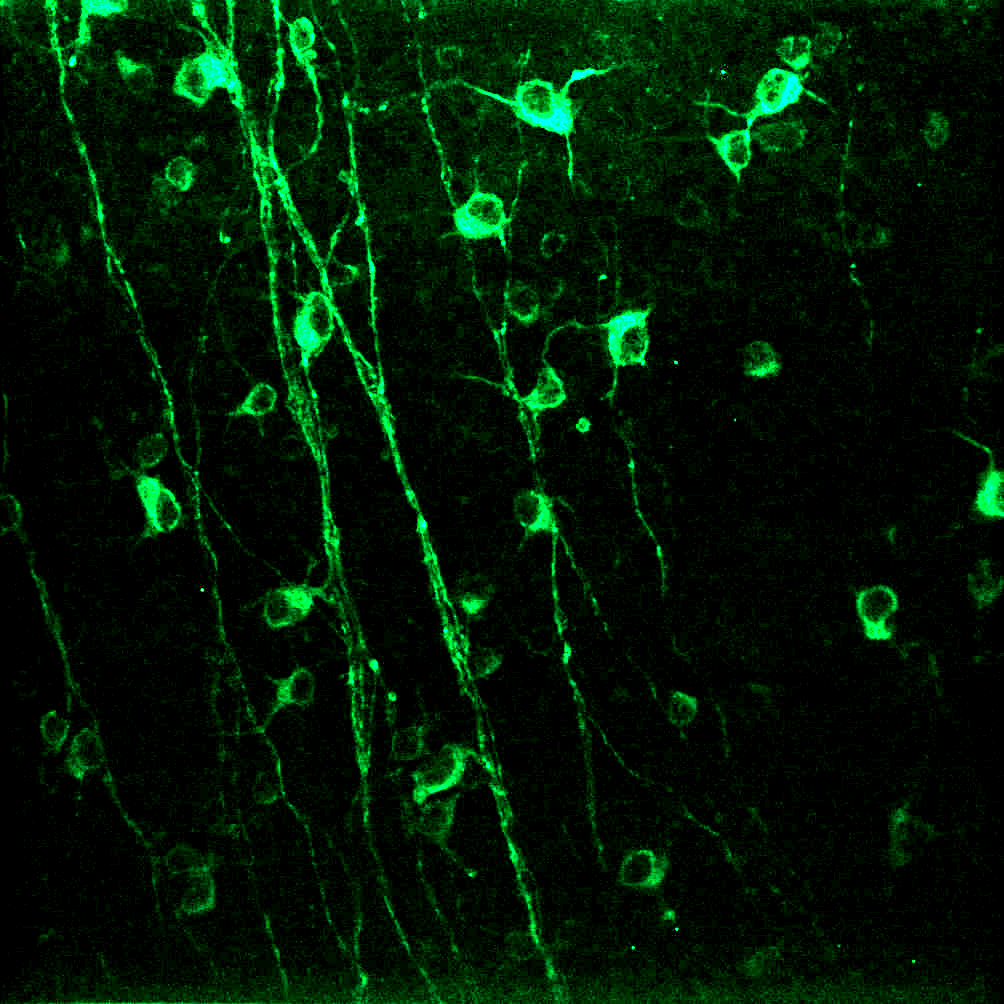

Supplement: Supplementary file 1 [file Data_Sheet_1.ZIP › Raw date/Figure 1/Original picture/eye drop/Drop 0.1%HupA_.jpg]

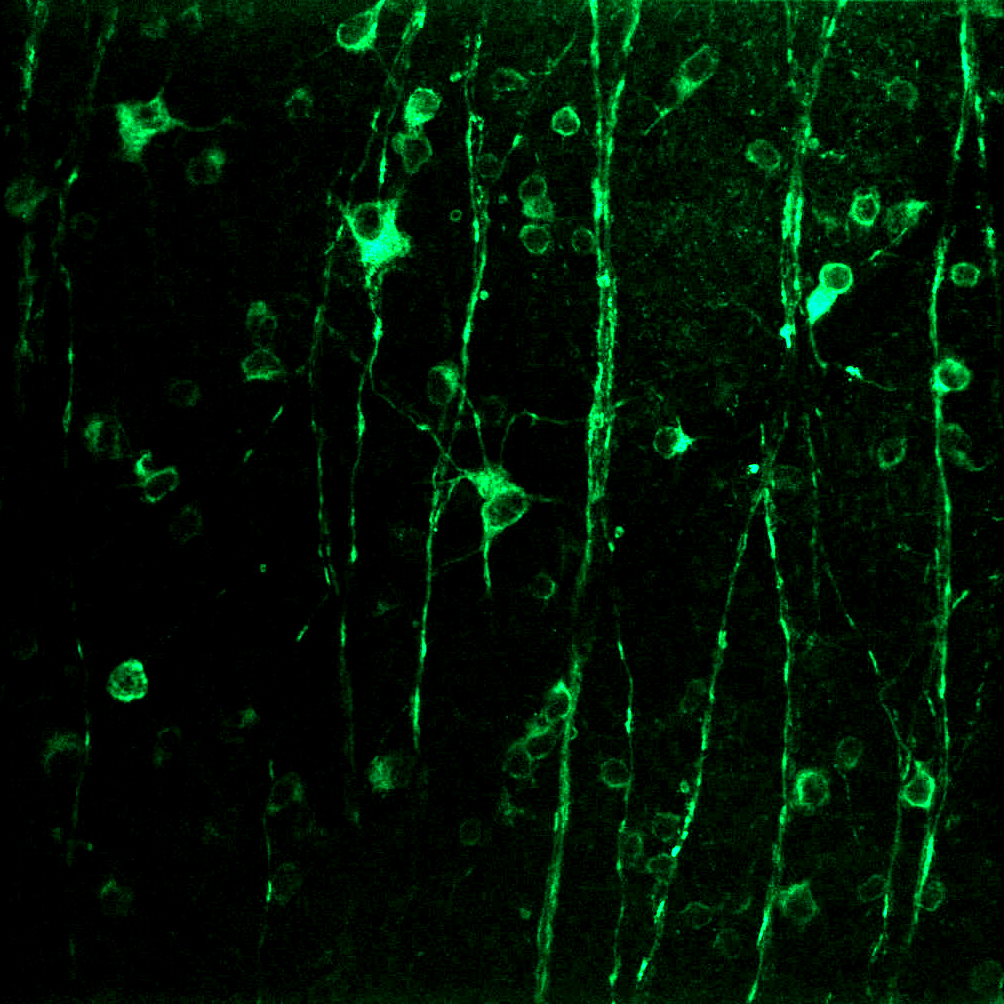

Supplement: Supplementary file 1 [file Data_Sheet_1.ZIP › Raw date/Figure 1/Original picture/Intravitreal injection/Inject 0.05%HupA.jpg]

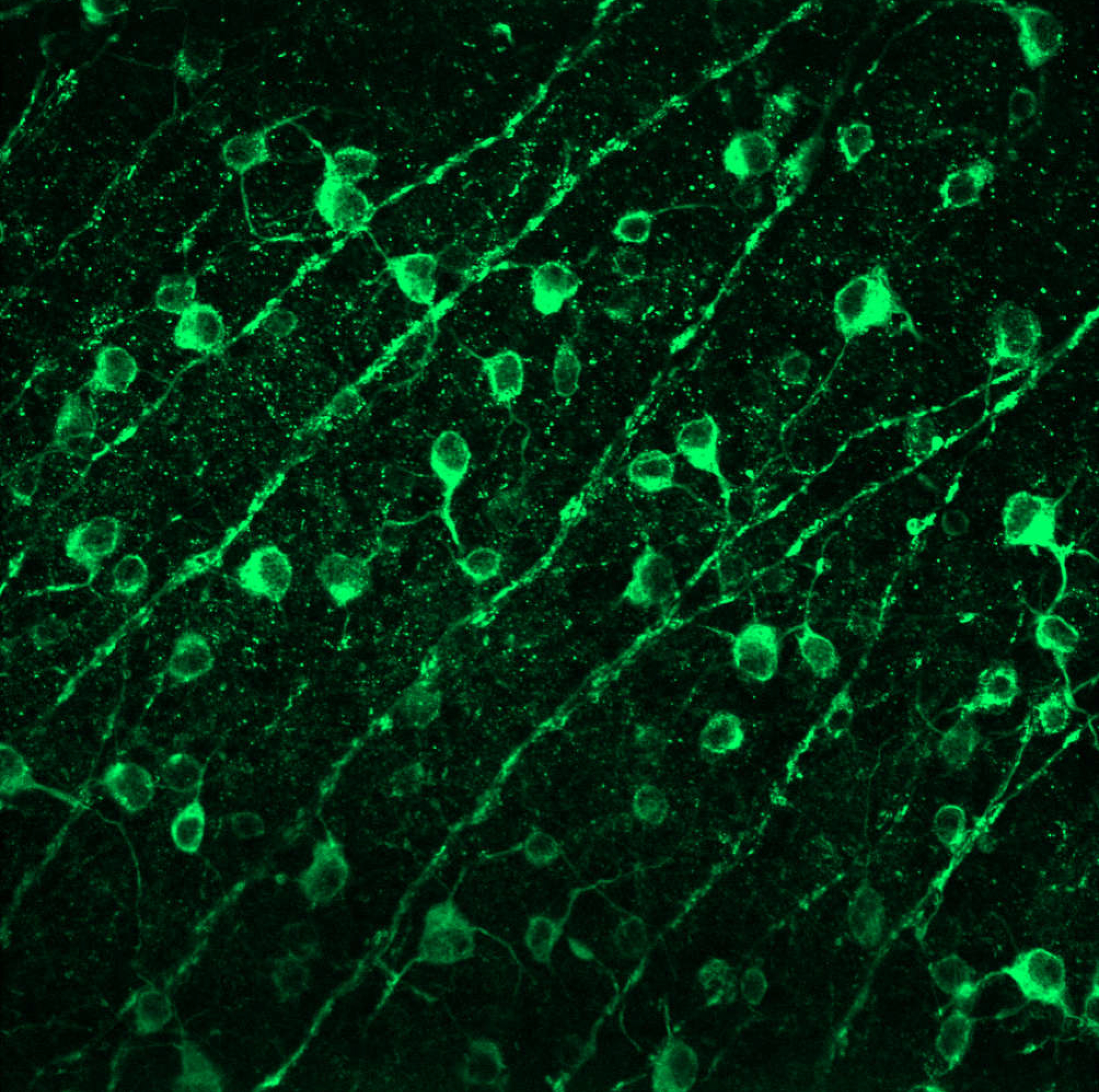

Supplement: Supplementary file 1 [file Data_Sheet_1.ZIP › Raw date/Figure 1/Original picture/Intravitreal injection/Inject 0.2%HupA.jpg]

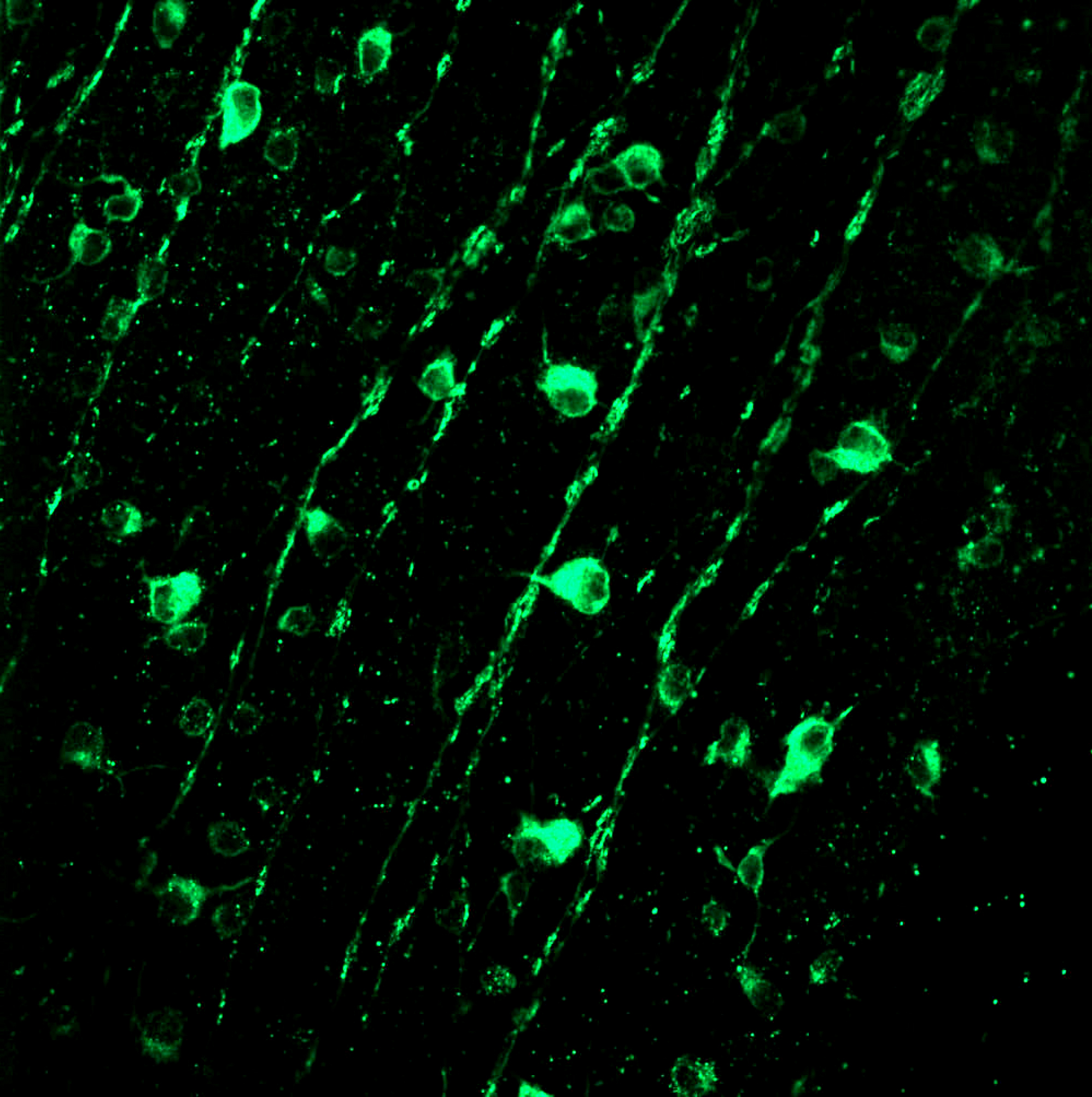

Supplement: Supplementary file 1 [file Data_Sheet_1.ZIP › Raw date/Figure 1/Original picture/Intravitreal injection/PBS.jpg]

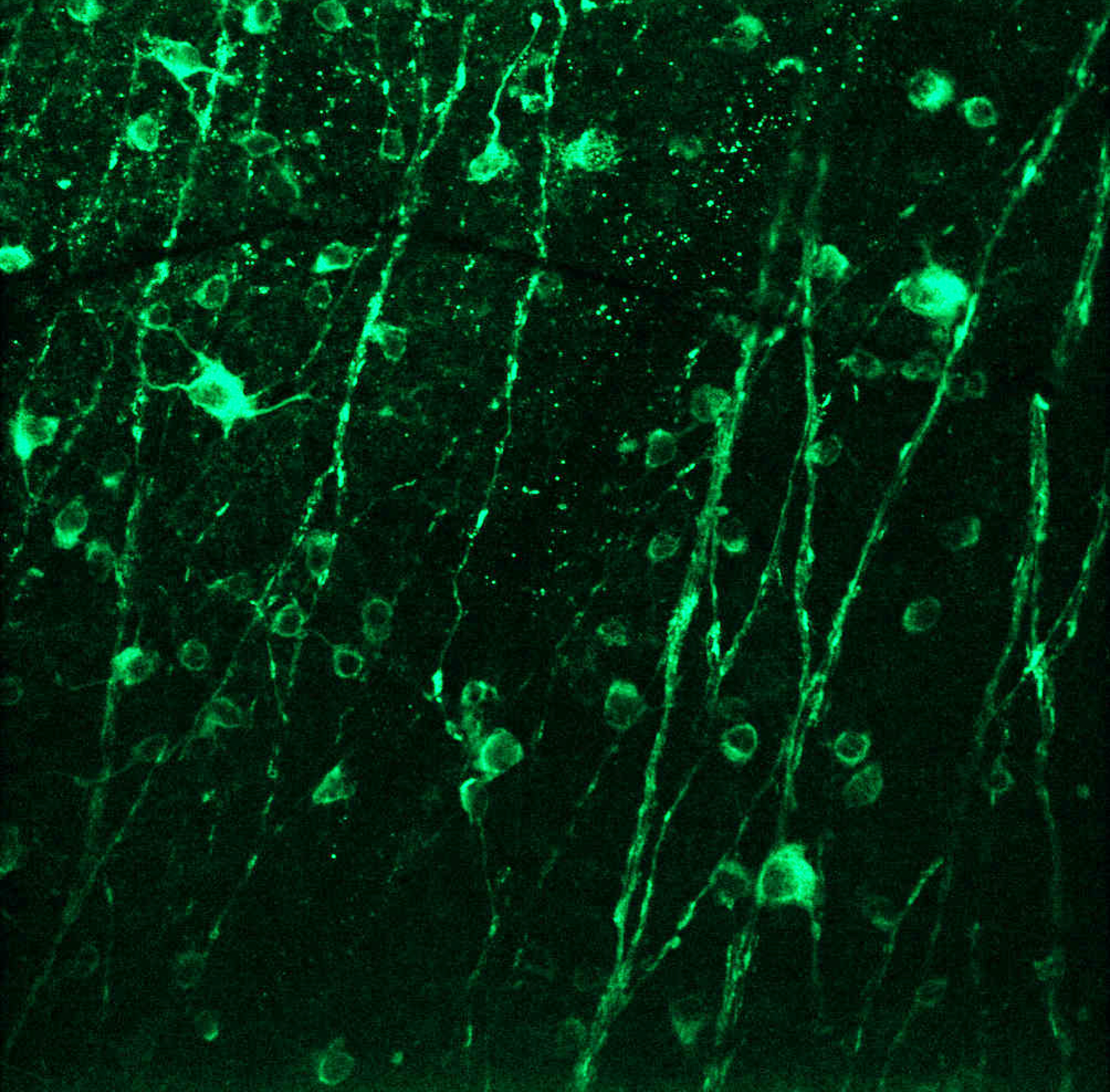

Supplement: Supplementary file 1 [file Data_Sheet_1.ZIP › Raw date/Figure 1/Original picture/Intravitreal injection/Inject 0.1%HupA.jpg]

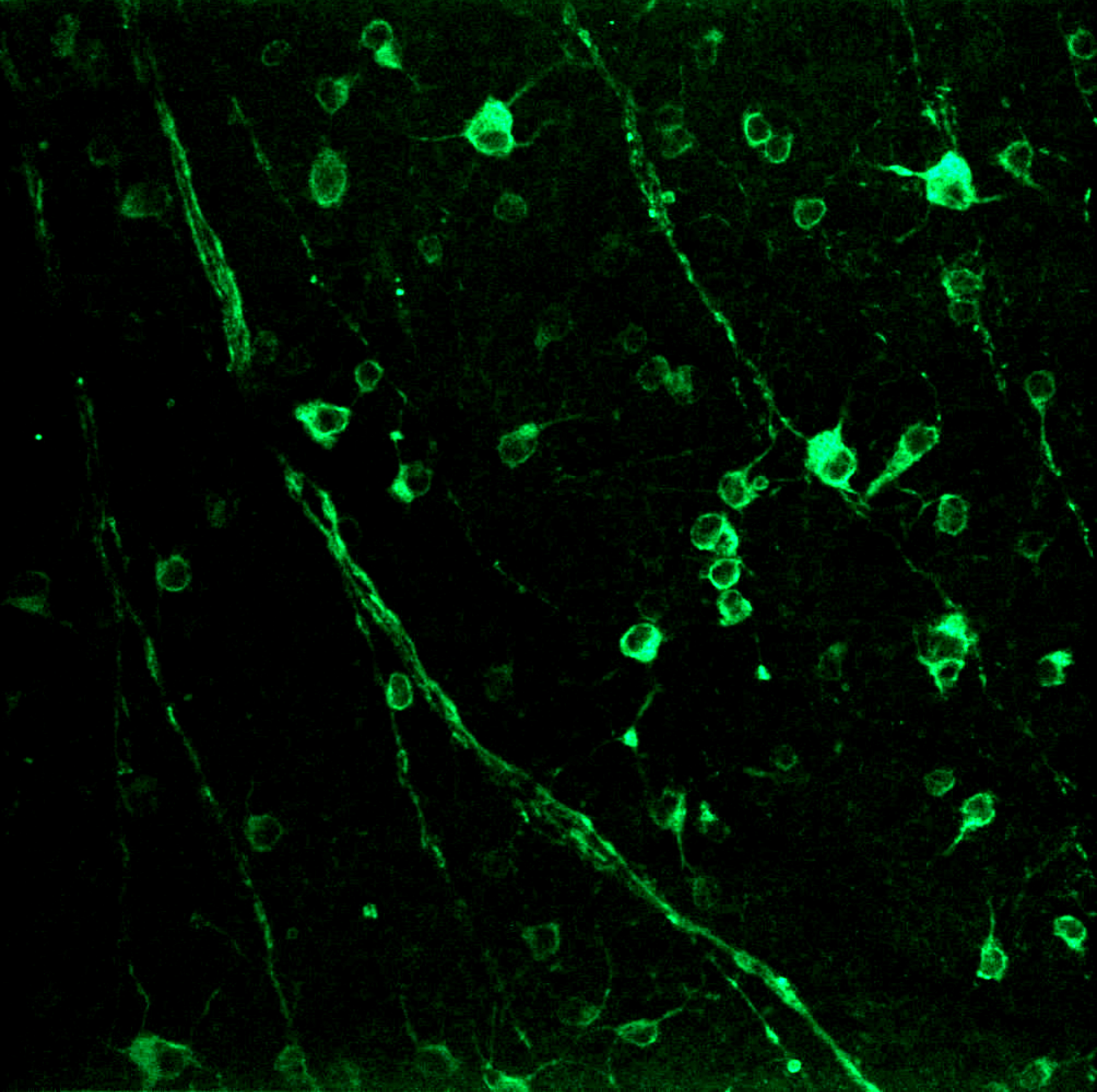

Supplement: Supplementary file 1 [file Data_Sheet_1.ZIP › Raw date/Figure 1/Original picture/Intraperitoneal injerction/PBS_.jpg]

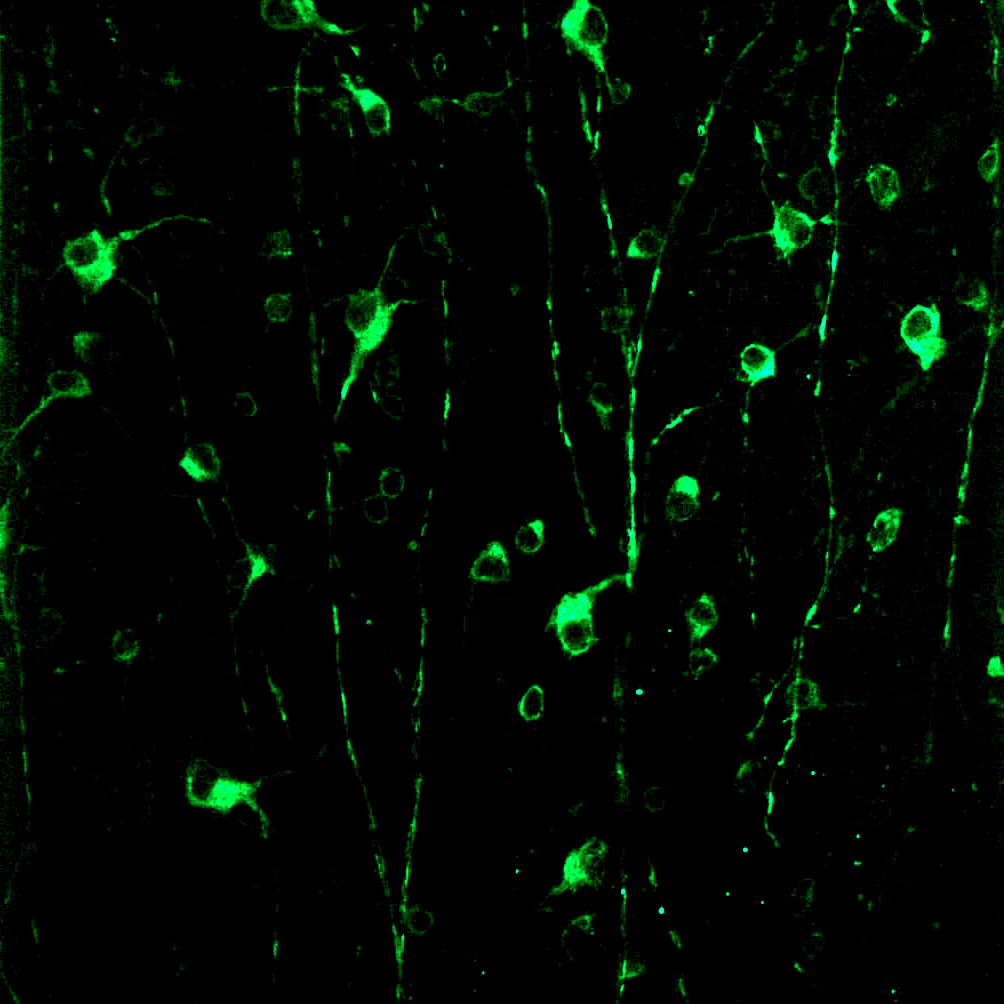

Supplement: Supplementary file 1 [file Data_Sheet_1.ZIP › Raw date/Figure 1/Original picture/Intraperitoneal injerction/0.2% HupA_.jpg]

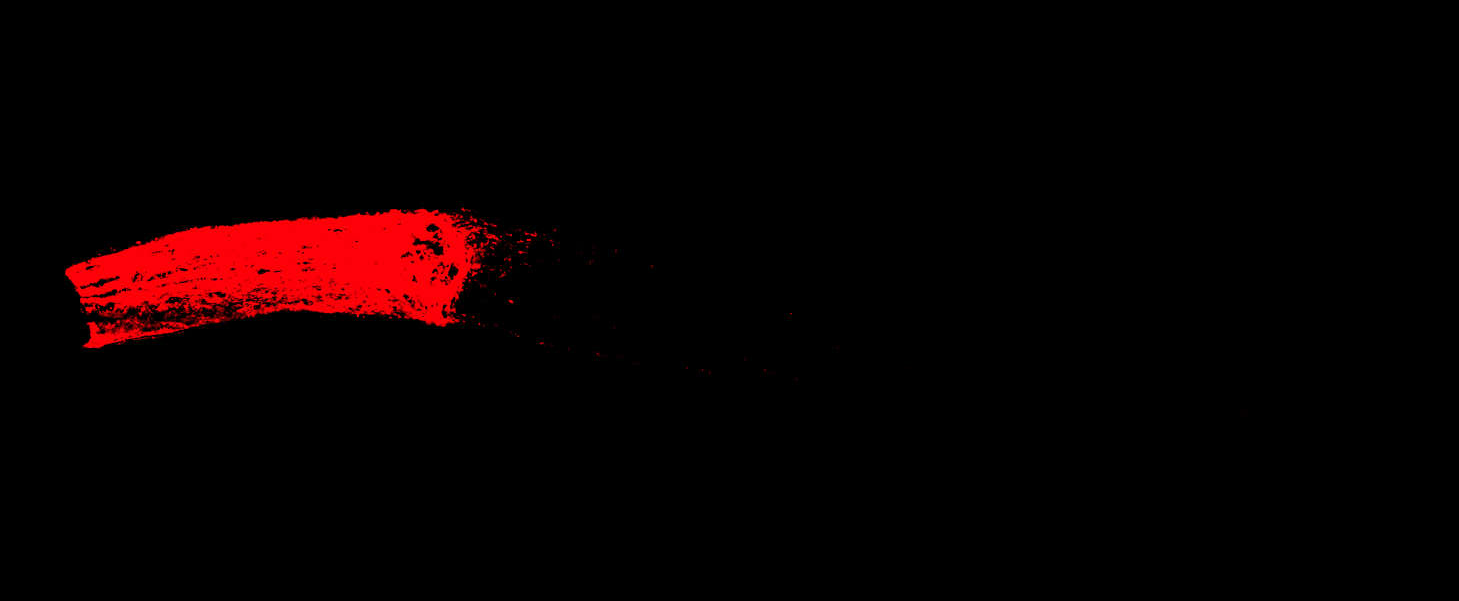

Supplement: Supplementary file 1 [file Data_Sheet_1.ZIP › Raw date/Figure 2/Original picture/Intravitreal injection/PBS.png]

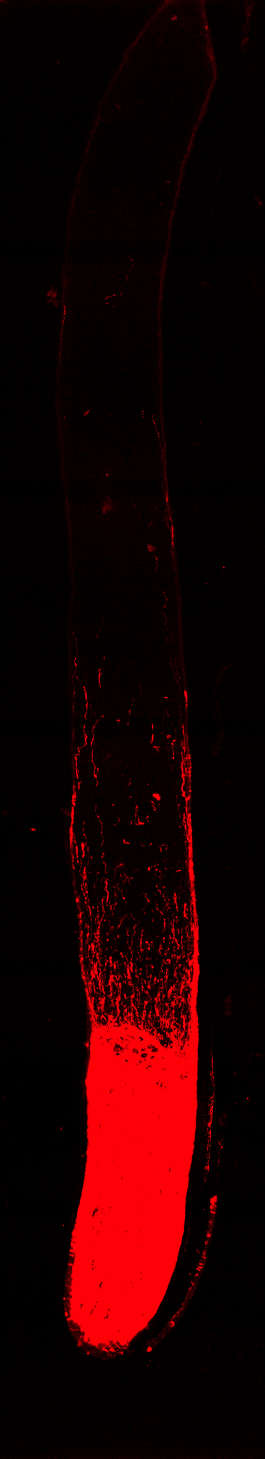

Supplement: Supplementary file 1 [file Data_Sheet_1.ZIP › Raw date/Figure 2/Original picture/Intravitreal injection/HupA.png]

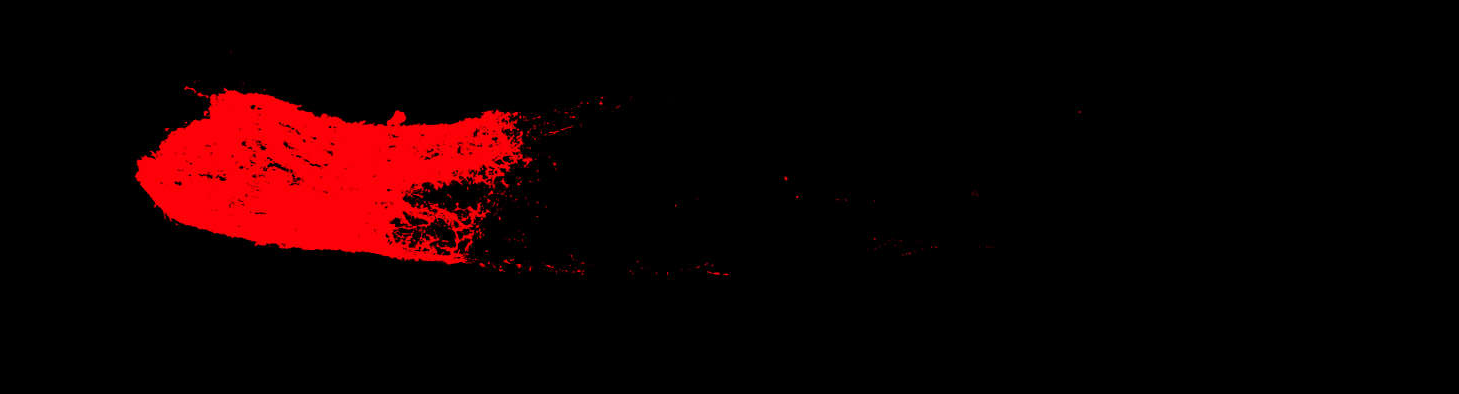

Supplement: Supplementary file 1 [file Data_Sheet_1.ZIP › Raw date/Figure 2/Original picture/Ocular drop/PBS .png]

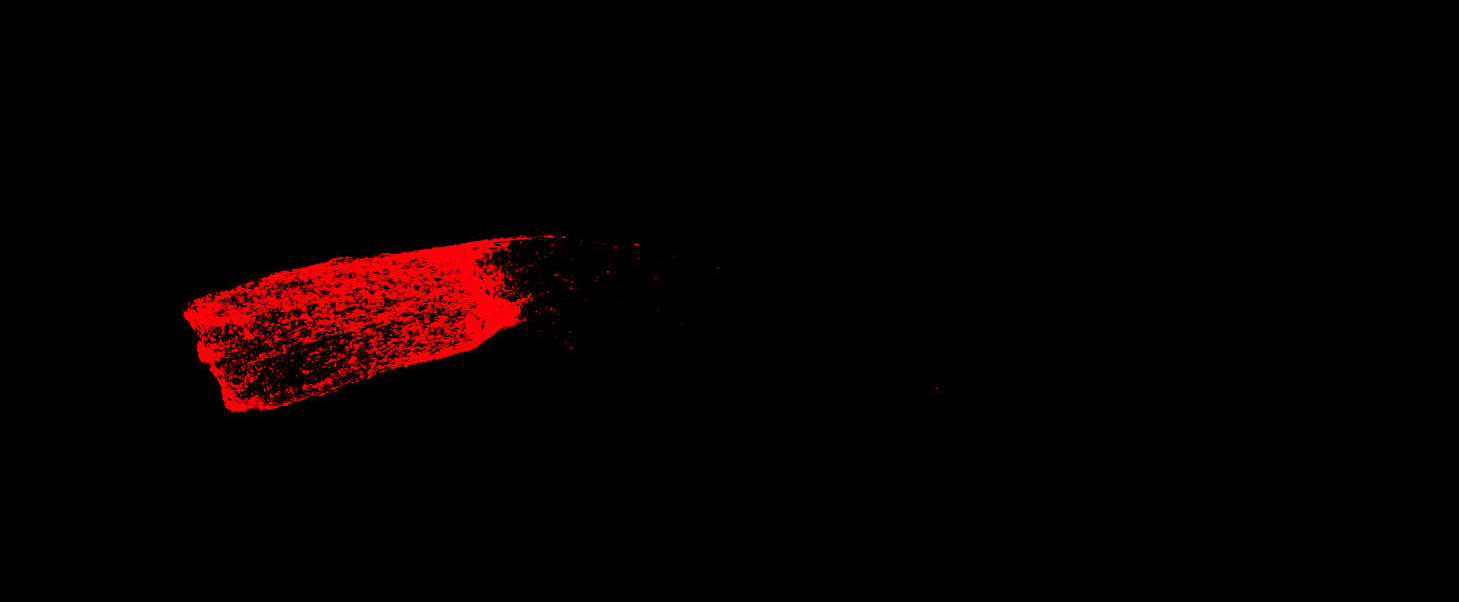

Supplement: Supplementary file 1 [file Data_Sheet_1.ZIP › Raw date/Figure 2/Original picture/Ocular drop/HupA.png]

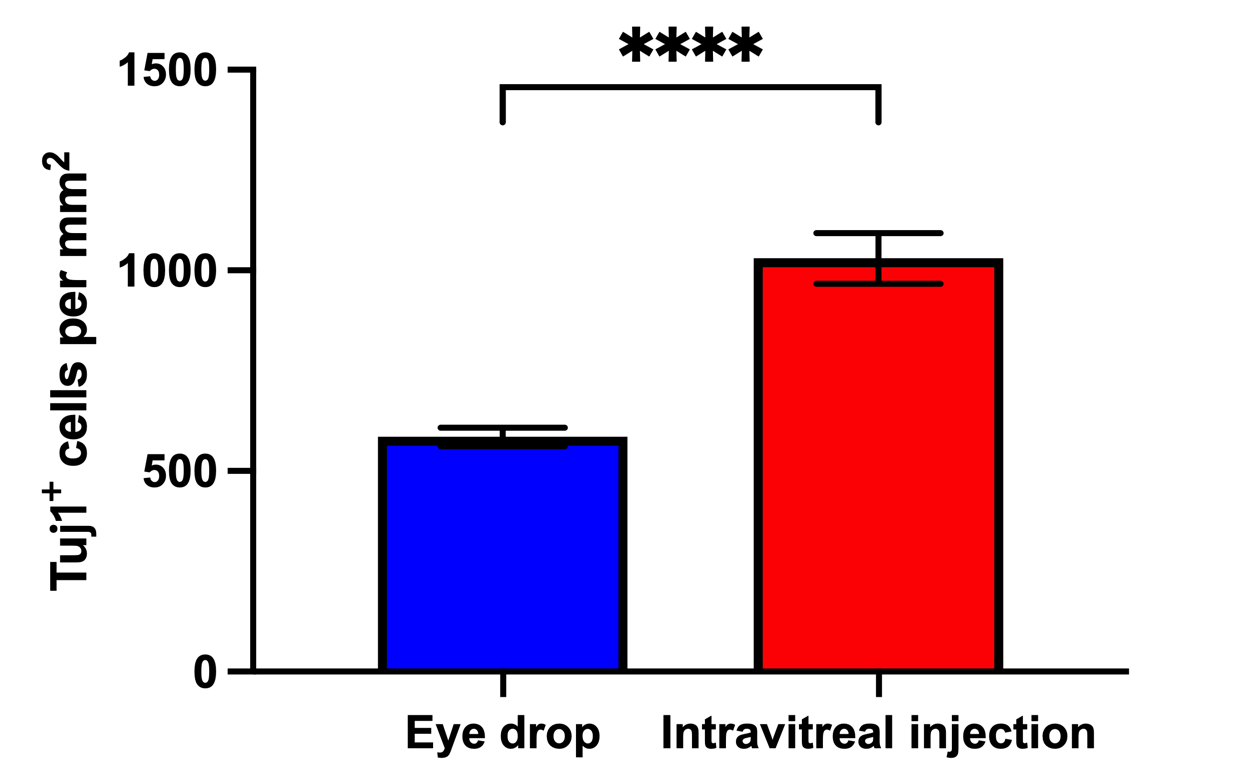

Supplement: Supplementary Figure 1 — Immunofluorescence staining of retinal sections at 14 days after ONC. (A) Representative confocal images of retinal sections showing surviving RBPMS RGCs using the ocular drip drug delivery method in the HupA-treatment and control mouse groups. Scale bar, 20 μm. (B) Quantification of the RGC survival rate in (A) (unpaired t-test, ****p < 0.0001; n = 5 mice in each group, at least eight non-adjacent retinal sections per mouse). (C) Representative confocal images of retinal sections showing surviving RBPMS RGCs using vitreous cavity injection administration in the HupA-treatment and control mouse groups. Scale bar, 20 μm. (D) Quantification of the RGC survival rate in (C) (Data are represented as mean ± SEM, unpaired t-test, ****p < 0.0001; n = 5 mice in each group, at least eight non-adjacent retinal sections per mouse). [file Data_Sheet_2.ZIP › Raw date(Supplementary material)/Figure S6/Fig S6 .tiff]

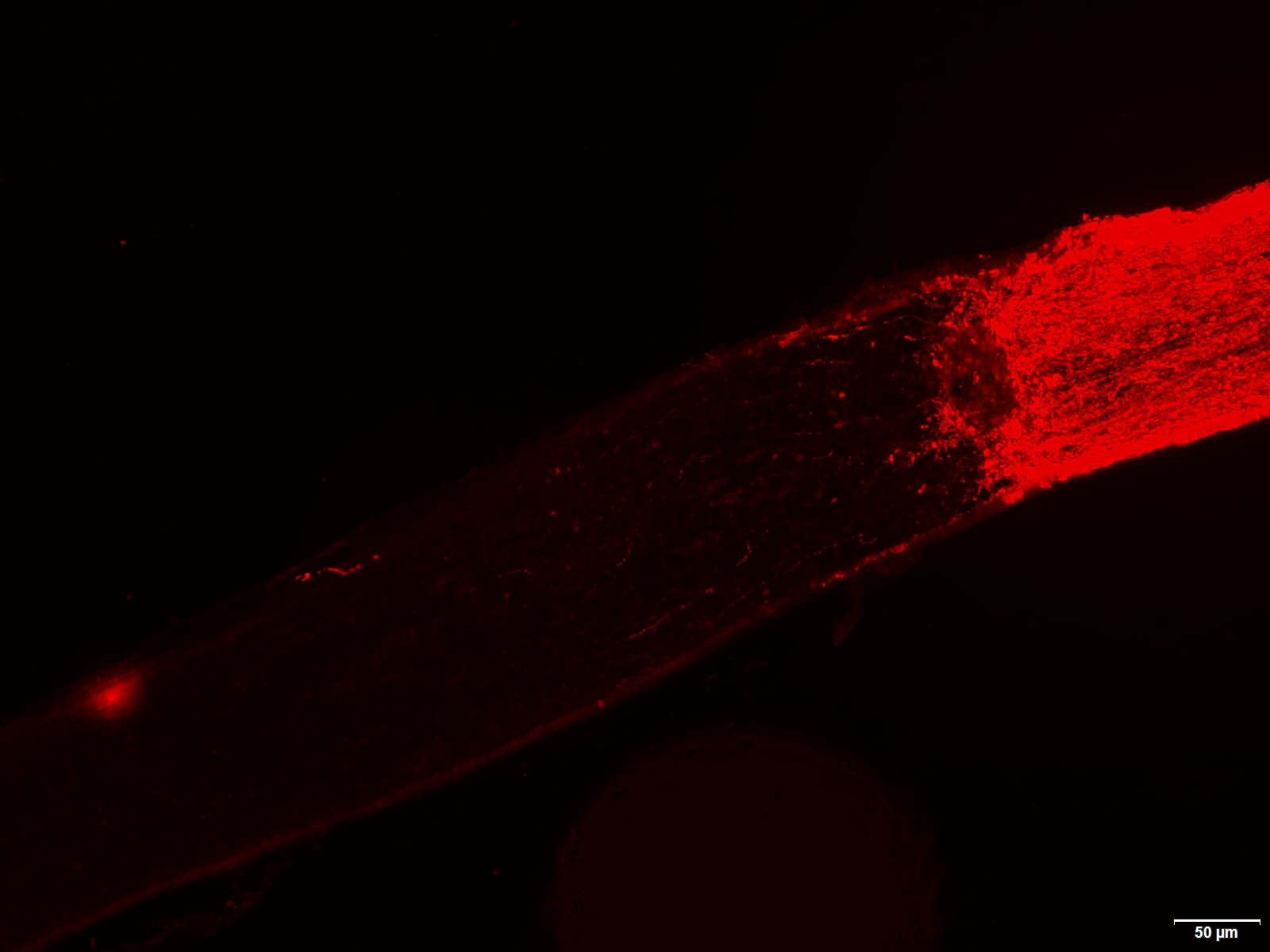

Supplement: Supplementary Figure 1 — Immunofluorescence staining of retinal sections at 14 days after ONC. (A) Representative confocal images of retinal sections showing surviving RBPMS RGCs using the ocular drip drug delivery method in the HupA-treatment and control mouse groups. Scale bar, 20 μm. (B) Quantification of the RGC survival rate in (A) (unpaired t-test, ****p < 0.0001; n = 5 mice in each group, at least eight non-adjacent retinal sections per mouse). (C) Representative confocal images of retinal sections showing surviving RBPMS RGCs using vitreous cavity injection administration in the HupA-treatment and control mouse groups. Scale bar, 20 μm. (D) Quantification of the RGC survival rate in (C) (Data are represented as mean ± SEM, unpaired t-test, ****p < 0.0001; n = 5 mice in each group, at least eight non-adjacent retinal sections per mouse). [file Data_Sheet_2.ZIP › Raw date(Supplementary material)/Figure S5/image/Rap.jpg]

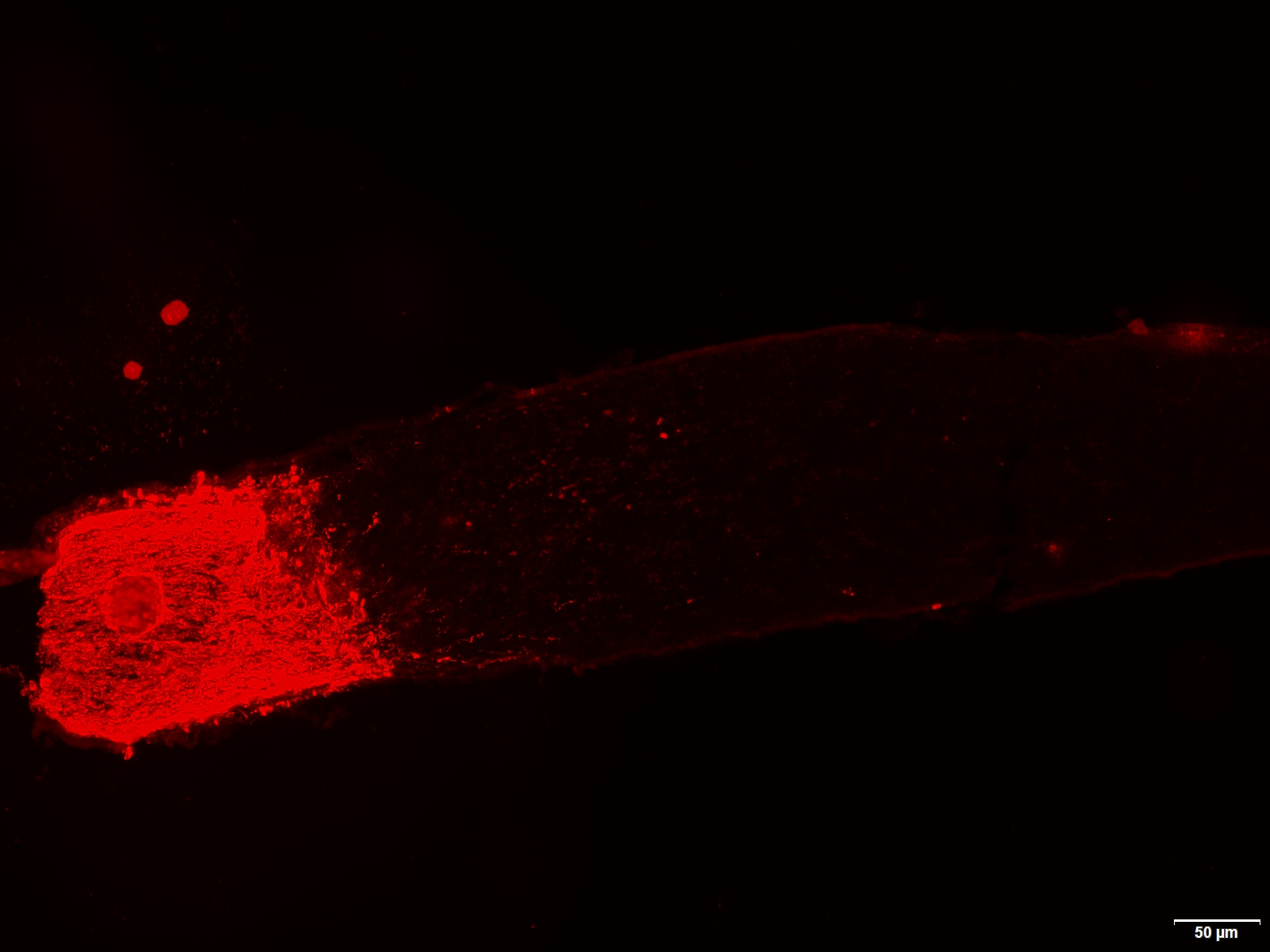

Supplement: Supplementary Figure 1 — Immunofluorescence staining of retinal sections at 14 days after ONC. (A) Representative confocal images of retinal sections showing surviving RBPMS RGCs using the ocular drip drug delivery method in the HupA-treatment and control mouse groups. Scale bar, 20 μm. (B) Quantification of the RGC survival rate in (A) (unpaired t-test, ****p < 0.0001; n = 5 mice in each group, at least eight non-adjacent retinal sections per mouse). (C) Representative confocal images of retinal sections showing surviving RBPMS RGCs using vitreous cavity injection administration in the HupA-treatment and control mouse groups. Scale bar, 20 μm. (D) Quantification of the RGC survival rate in (C) (Data are represented as mean ± SEM, unpaired t-test, ****p < 0.0001; n = 5 mice in each group, at least eight non-adjacent retinal sections per mouse). [file Data_Sheet_2.ZIP › Raw date(Supplementary material)/Figure S5/image/PBS.jpg]

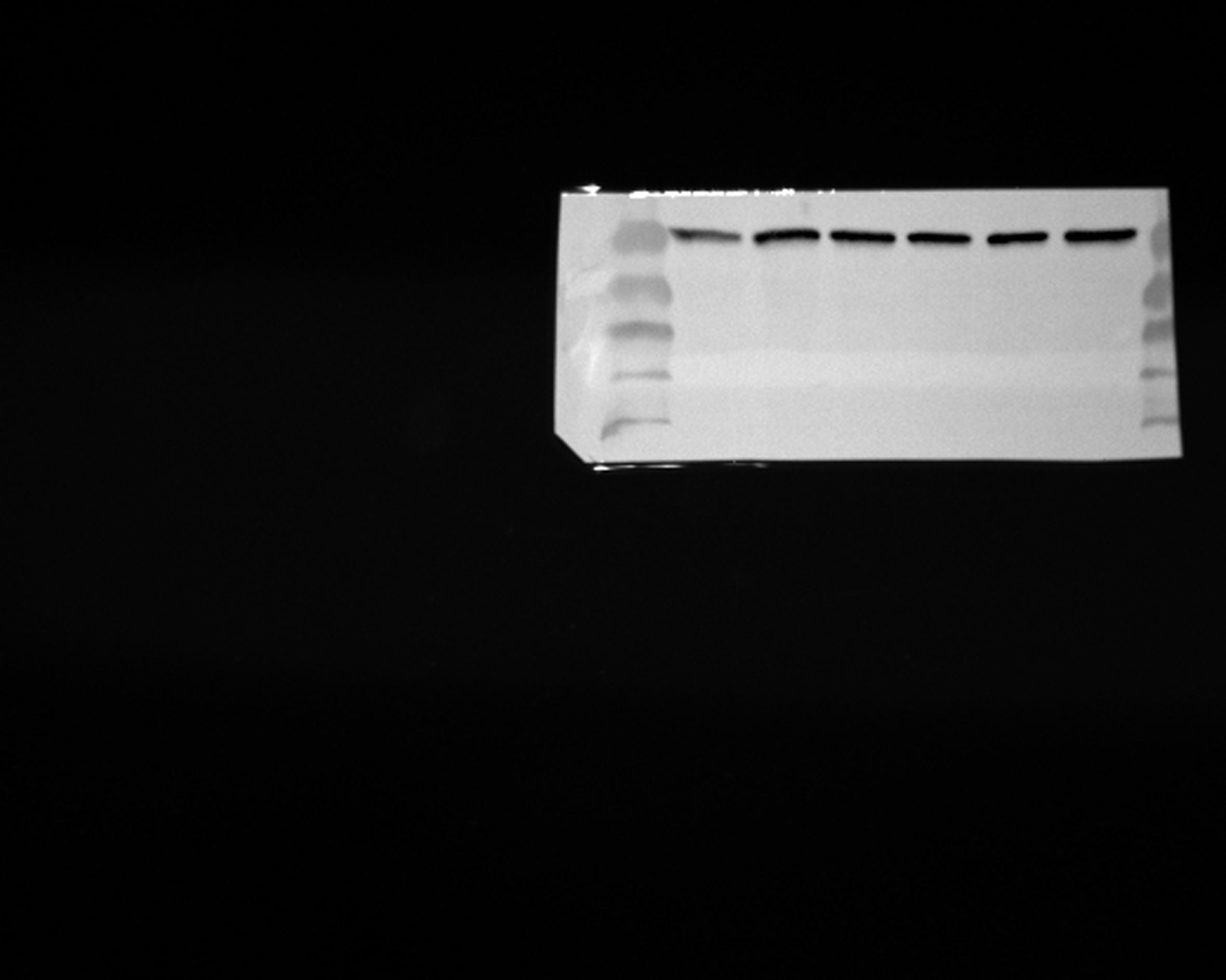

Supplement: Supplementary Figure 1 — Immunofluorescence staining of retinal sections at 14 days after ONC. (A) Representative confocal images of retinal sections showing surviving RBPMS RGCs using the ocular drip drug delivery method in the HupA-treatment and control mouse groups. Scale bar, 20 μm. (B) Quantification of the RGC survival rate in (A) (unpaired t-test, ****p < 0.0001; n = 5 mice in each group, at least eight non-adjacent retinal sections per mouse). (C) Representative confocal images of retinal sections showing surviving RBPMS RGCs using vitreous cavity injection administration in the HupA-treatment and control mouse groups. Scale bar, 20 μm. (D) Quantification of the RGC survival rate in (C) (Data are represented as mean ± SEM, unpaired t-test, ****p < 0.0001; n = 5 mice in each group, at least eight non-adjacent retinal sections per mouse). [file Data_Sheet_2.ZIP › Raw date(Supplementary material)/Figure S2/image/tubulin 55kd .tif]

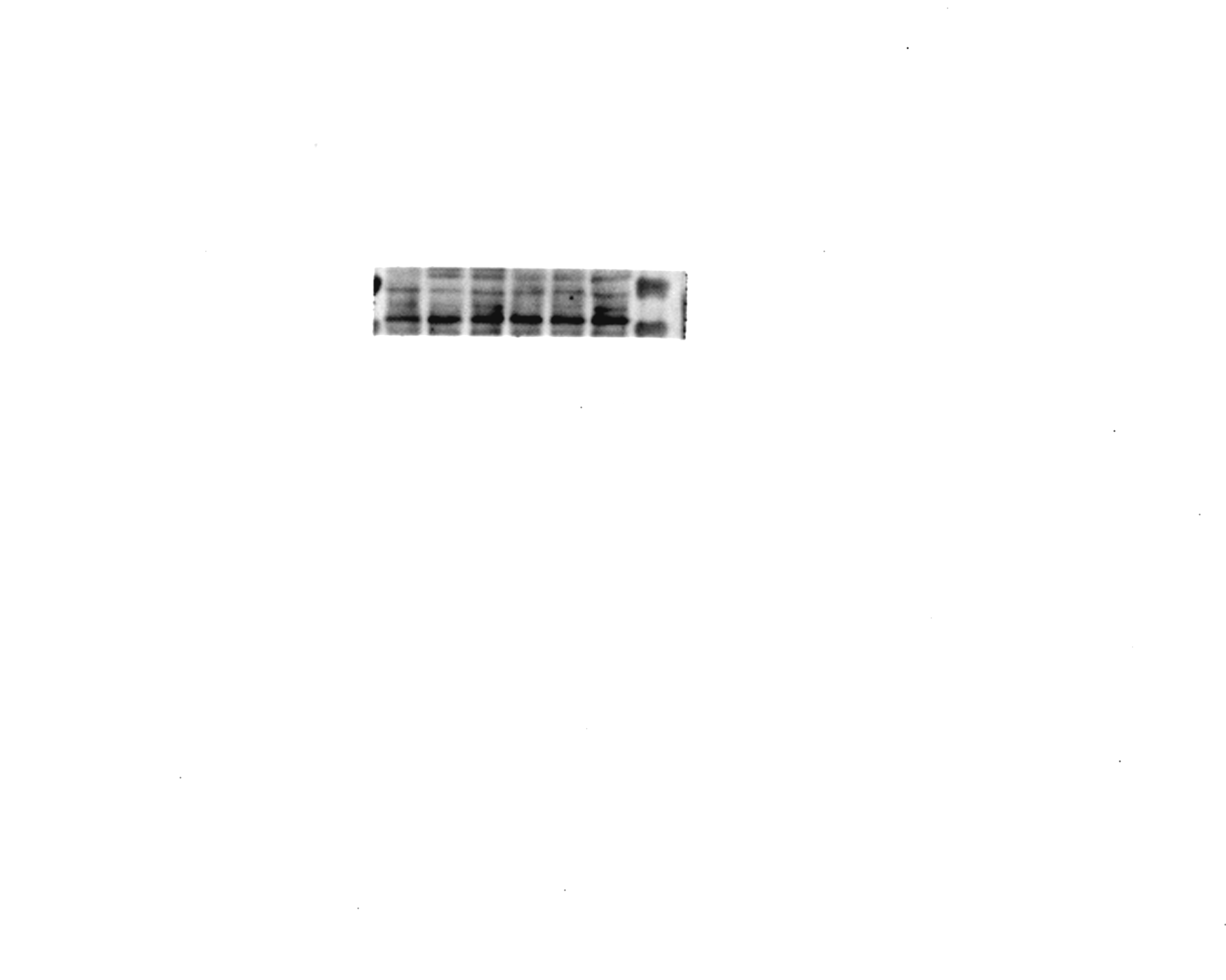

Supplement: Supplementary Figure 1 — Immunofluorescence staining of retinal sections at 14 days after ONC. (A) Representative confocal images of retinal sections showing surviving RBPMS RGCs using the ocular drip drug delivery method in the HupA-treatment and control mouse groups. Scale bar, 20 μm. (B) Quantification of the RGC survival rate in (A) (unpaired t-test, ****p < 0.0001; n = 5 mice in each group, at least eight non-adjacent retinal sections per mouse). (C) Representative confocal images of retinal sections showing surviving RBPMS RGCs using vitreous cavity injection administration in the HupA-treatment and control mouse groups. Scale bar, 20 μm. (D) Quantification of the RGC survival rate in (C) (Data are represented as mean ± SEM, unpaired t-test, ****p < 0.0001; n = 5 mice in each group, at least eight non-adjacent retinal sections per mouse). [file Data_Sheet_2.ZIP › Raw date(Supplementary material)/Figure S2/image/s6.tif]

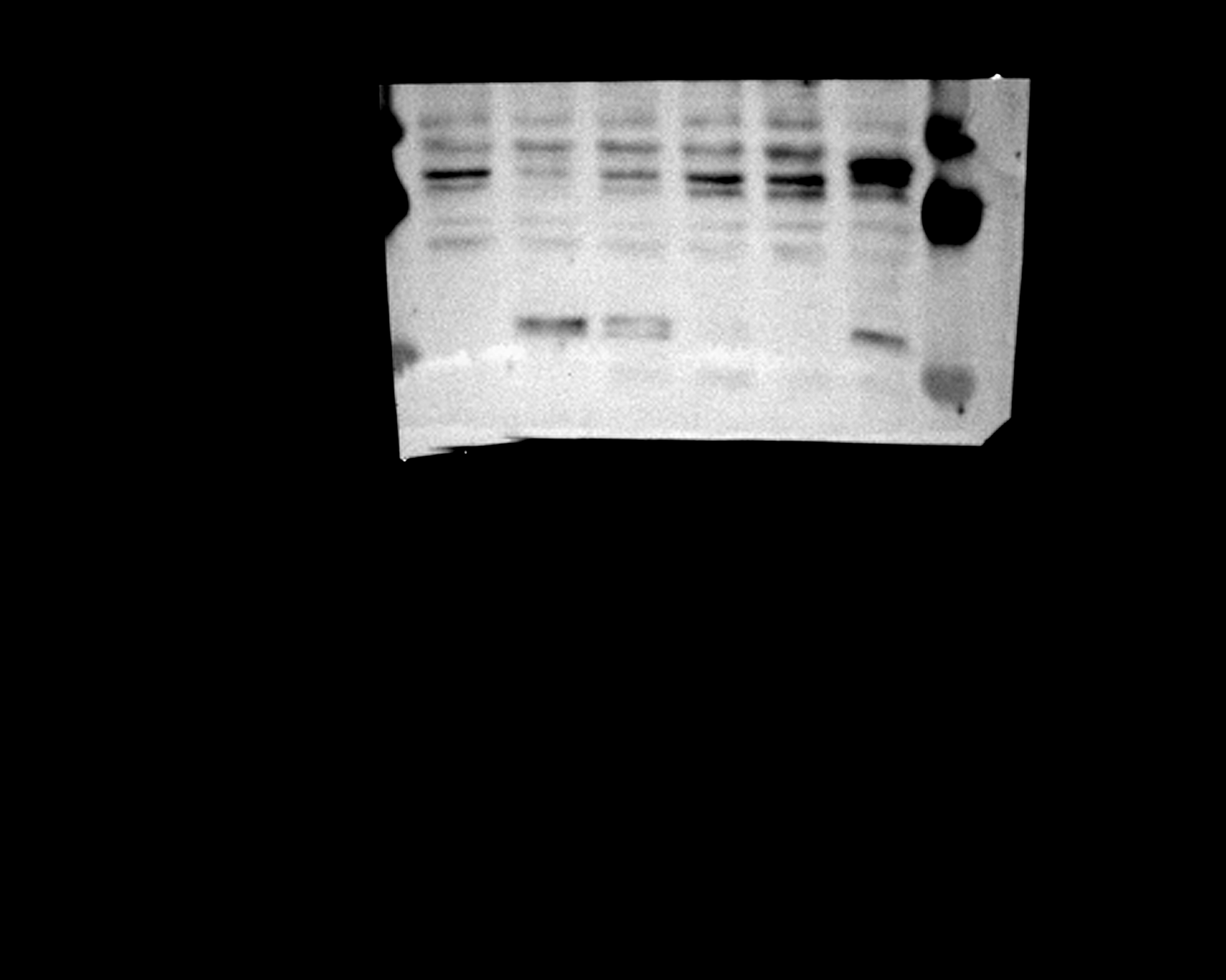

Supplement: Supplementary Figure 1 — Immunofluorescence staining of retinal sections at 14 days after ONC. (A) Representative confocal images of retinal sections showing surviving RBPMS RGCs using the ocular drip drug delivery method in the HupA-treatment and control mouse groups. Scale bar, 20 μm. (B) Quantification of the RGC survival rate in (A) (unpaired t-test, ****p < 0.0001; n = 5 mice in each group, at least eight non-adjacent retinal sections per mouse). (C) Representative confocal images of retinal sections showing surviving RBPMS RGCs using vitreous cavity injection administration in the HupA-treatment and control mouse groups. Scale bar, 20 μm. (D) Quantification of the RGC survival rate in (C) (Data are represented as mean ± SEM, unpaired t-test, ****p < 0.0001; n = 5 mice in each group, at least eight non-adjacent retinal sections per mouse). [file Data_Sheet_2.ZIP › Raw date(Supplementary material)/Figure S2/image/PS6.tif]

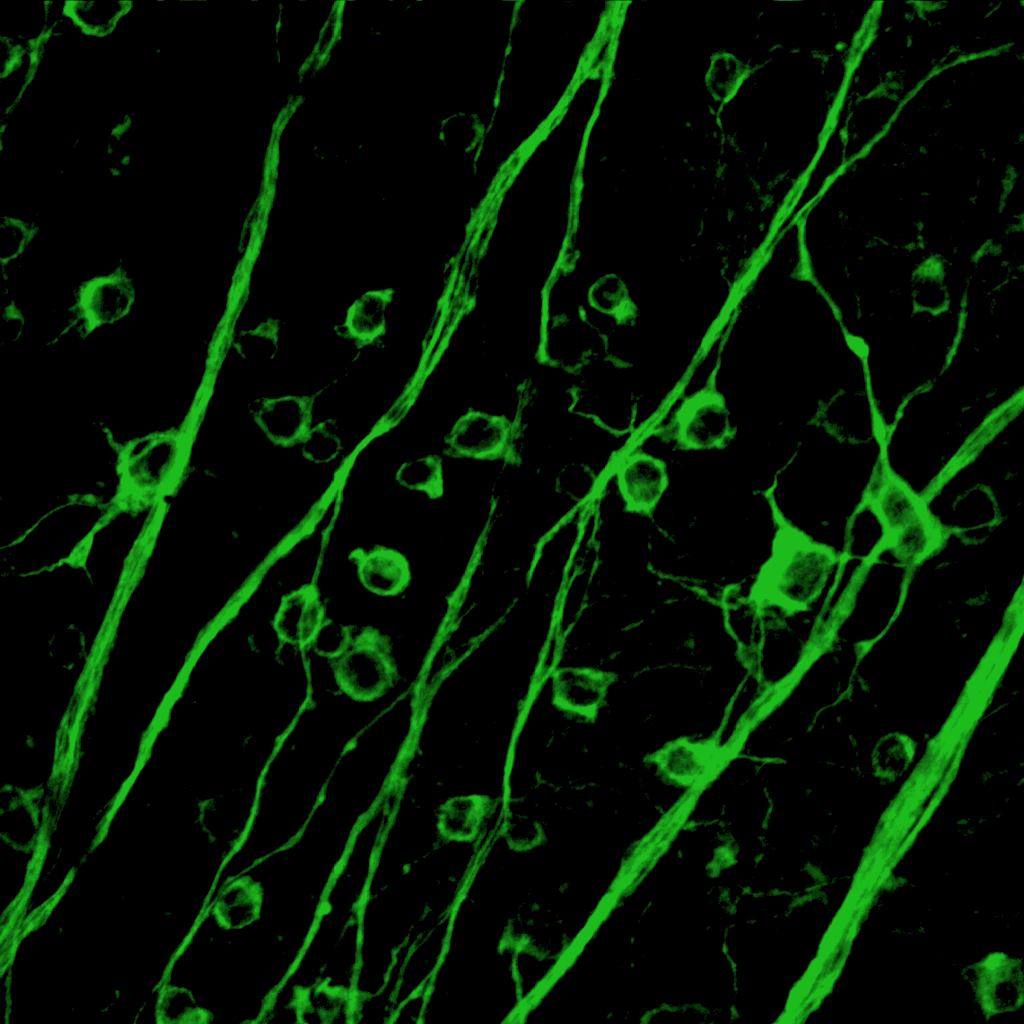

Supplement: Supplementary Figure 1 — Immunofluorescence staining of retinal sections at 14 days after ONC. (A) Representative confocal images of retinal sections showing surviving RBPMS RGCs using the ocular drip drug delivery method in the HupA-treatment and control mouse groups. Scale bar, 20 μm. (B) Quantification of the RGC survival rate in (A) (unpaired t-test, ****p < 0.0001; n = 5 mice in each group, at least eight non-adjacent retinal sections per mouse). (C) Representative confocal images of retinal sections showing surviving RBPMS RGCs using vitreous cavity injection administration in the HupA-treatment and control mouse groups. Scale bar, 20 μm. (D) Quantification of the RGC survival rate in (C) (Data are represented as mean ± SEM, unpaired t-test, ****p < 0.0001; n = 5 mice in each group, at least eight non-adjacent retinal sections per mouse). [file Data_Sheet_2.ZIP › Raw date(Supplementary material)/Figure S7/image/6mg:kg Rap.tif]

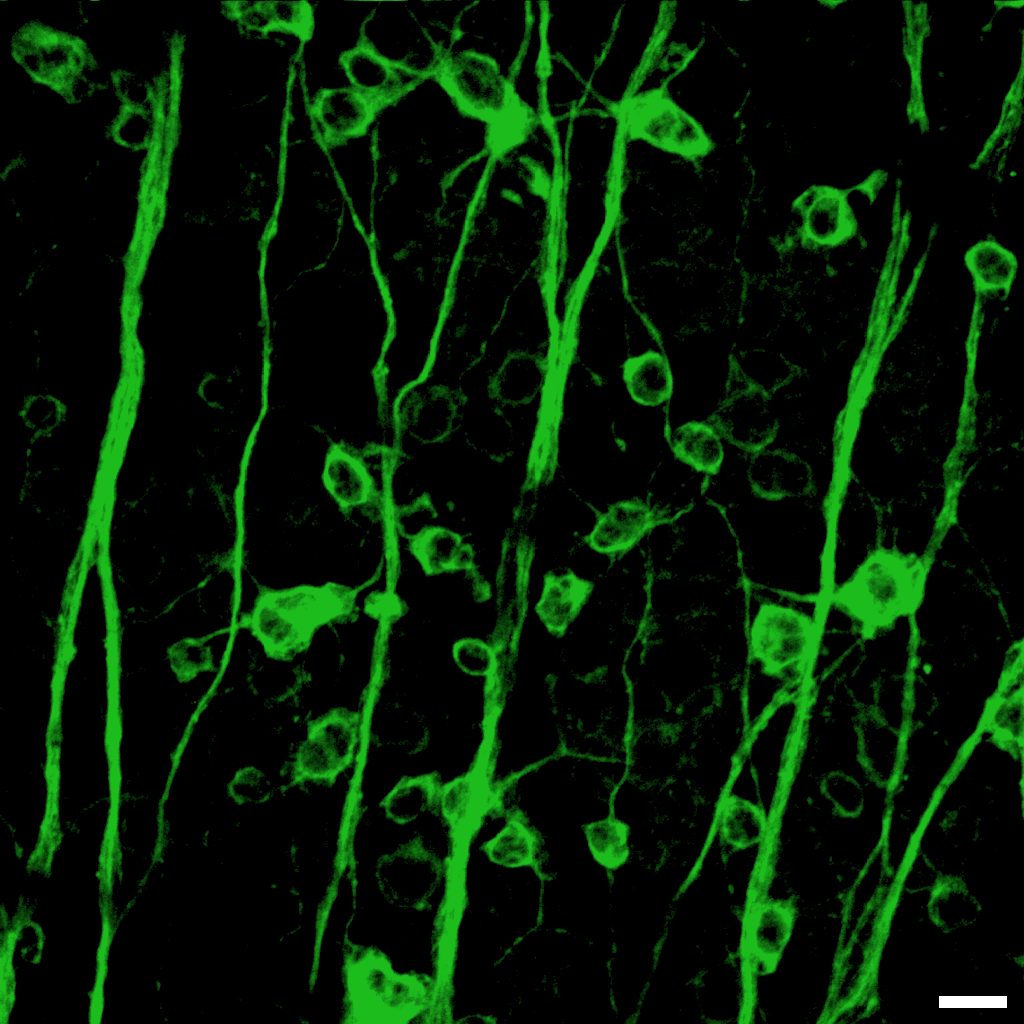

Supplement: Supplementary Figure 1 — Immunofluorescence staining of retinal sections at 14 days after ONC. (A) Representative confocal images of retinal sections showing surviving RBPMS RGCs using the ocular drip drug delivery method in the HupA-treatment and control mouse groups. Scale bar, 20 μm. (B) Quantification of the RGC survival rate in (A) (unpaired t-test, ****p < 0.0001; n = 5 mice in each group, at least eight non-adjacent retinal sections per mouse). (C) Representative confocal images of retinal sections showing surviving RBPMS RGCs using vitreous cavity injection administration in the HupA-treatment and control mouse groups. Scale bar, 20 μm. (D) Quantification of the RGC survival rate in (C) (Data are represented as mean ± SEM, unpaired t-test, ****p < 0.0001; n = 5 mice in each group, at least eight non-adjacent retinal sections per mouse). [file Data_Sheet_2.ZIP › Raw date(Supplementary material)/Figure S7/image/7.5mg:kg Rap.tif]

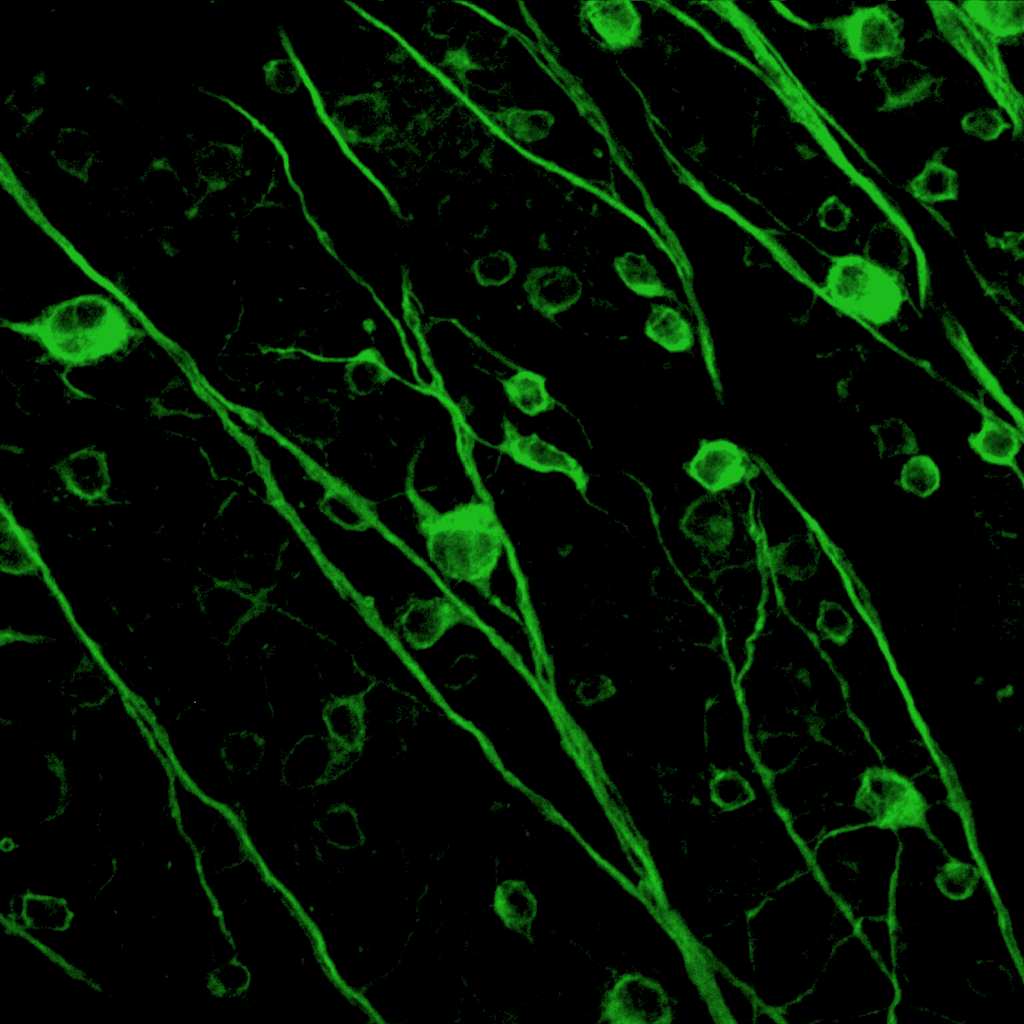

Supplement: Supplementary Figure 1 — Immunofluorescence staining of retinal sections at 14 days after ONC. (A) Representative confocal images of retinal sections showing surviving RBPMS RGCs using the ocular drip drug delivery method in the HupA-treatment and control mouse groups. Scale bar, 20 μm. (B) Quantification of the RGC survival rate in (A) (unpaired t-test, ****p < 0.0001; n = 5 mice in each group, at least eight non-adjacent retinal sections per mouse). (C) Representative confocal images of retinal sections showing surviving RBPMS RGCs using vitreous cavity injection administration in the HupA-treatment and control mouse groups. Scale bar, 20 μm. (D) Quantification of the RGC survival rate in (C) (Data are represented as mean ± SEM, unpaired t-test, ****p < 0.0001; n = 5 mice in each group, at least eight non-adjacent retinal sections per mouse). [file Data_Sheet_2.ZIP › Raw date(Supplementary material)/Figure S7/image/PBS.tif]

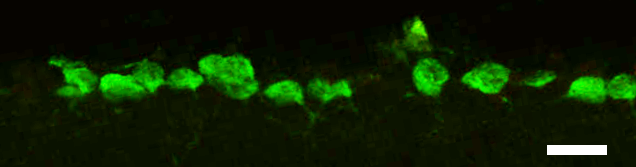

Supplement: Supplementary Figure 1 — Immunofluorescence staining of retinal sections at 14 days after ONC. (A) Representative confocal images of retinal sections showing surviving RBPMS RGCs using the ocular drip drug delivery method in the HupA-treatment and control mouse groups. Scale bar, 20 μm. (B) Quantification of the RGC survival rate in (A) (unpaired t-test, ****p < 0.0001; n = 5 mice in each group, at least eight non-adjacent retinal sections per mouse). (C) Representative confocal images of retinal sections showing surviving RBPMS RGCs using vitreous cavity injection administration in the HupA-treatment and control mouse groups. Scale bar, 20 μm. (D) Quantification of the RGC survival rate in (C) (Data are represented as mean ± SEM, unpaired t-test, ****p < 0.0001; n = 5 mice in each group, at least eight non-adjacent retinal sections per mouse). [file Data_Sheet_2.ZIP › Raw date(Supplementary material)/Figure S4/image/Rap/bar11.tif]

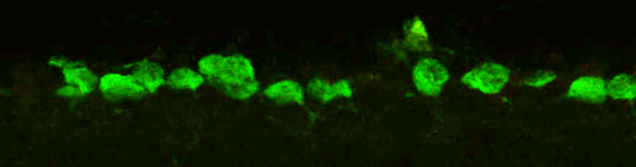

Supplement: Supplementary Figure 1 — Immunofluorescence staining of retinal sections at 14 days after ONC. (A) Representative confocal images of retinal sections showing surviving RBPMS RGCs using the ocular drip drug delivery method in the HupA-treatment and control mouse groups. Scale bar, 20 μm. (B) Quantification of the RGC survival rate in (A) (unpaired t-test, ****p < 0.0001; n = 5 mice in each group, at least eight non-adjacent retinal sections per mouse). (C) Representative confocal images of retinal sections showing surviving RBPMS RGCs using vitreous cavity injection administration in the HupA-treatment and control mouse groups. Scale bar, 20 μm. (D) Quantification of the RGC survival rate in (C) (Data are represented as mean ± SEM, unpaired t-test, ****p < 0.0001; n = 5 mice in each group, at least eight non-adjacent retinal sections per mouse). [file Data_Sheet_2.ZIP › Raw date(Supplementary material)/Figure S4/image/Rap/11.tif (RGB).tif]

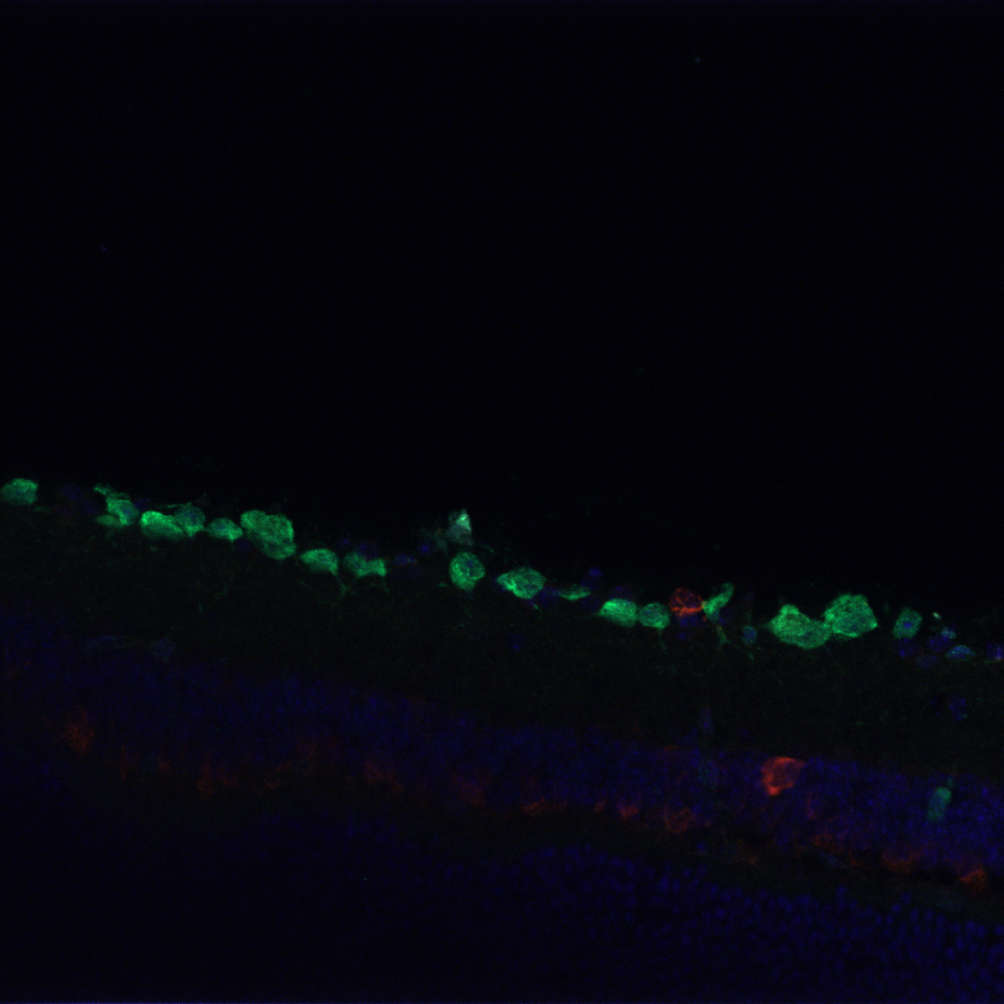

Supplement: Supplementary Figure 1 — Immunofluorescence staining of retinal sections at 14 days after ONC. (A) Representative confocal images of retinal sections showing surviving RBPMS RGCs using the ocular drip drug delivery method in the HupA-treatment and control mouse groups. Scale bar, 20 μm. (B) Quantification of the RGC survival rate in (A) (unpaired t-test, ****p < 0.0001; n = 5 mice in each group, at least eight non-adjacent retinal sections per mouse). (C) Representative confocal images of retinal sections showing surviving RBPMS RGCs using vitreous cavity injection administration in the HupA-treatment and control mouse groups. Scale bar, 20 μm. (D) Quantification of the RGC survival rate in (C) (Data are represented as mean ± SEM, unpaired t-test, ****p < 0.0001; n = 5 mice in each group, at least eight non-adjacent retinal sections per mouse). [file Data_Sheet_2.ZIP › Raw date(Supplementary material)/Figure S4/image/Rap/Rap.png]

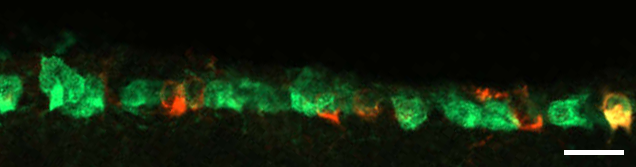

Supplement: Supplementary Figure 1 — Immunofluorescence staining of retinal sections at 14 days after ONC. (A) Representative confocal images of retinal sections showing surviving RBPMS RGCs using the ocular drip drug delivery method in the HupA-treatment and control mouse groups. Scale bar, 20 μm. (B) Quantification of the RGC survival rate in (A) (unpaired t-test, ****p < 0.0001; n = 5 mice in each group, at least eight non-adjacent retinal sections per mouse). (C) Representative confocal images of retinal sections showing surviving RBPMS RGCs using vitreous cavity injection administration in the HupA-treatment and control mouse groups. Scale bar, 20 μm. (D) Quantification of the RGC survival rate in (C) (Data are represented as mean ± SEM, unpaired t-test, ****p < 0.0001; n = 5 mice in each group, at least eight non-adjacent retinal sections per mouse). [file Data_Sheet_2.ZIP › Raw date(Supplementary material)/Figure S4/image/PBS/bar11.tif]

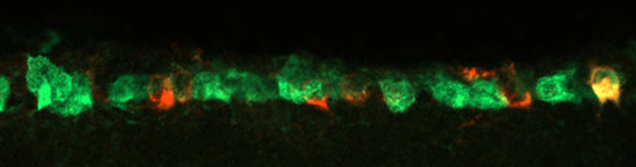

Supplement: Supplementary Figure 1 — Immunofluorescence staining of retinal sections at 14 days after ONC. (A) Representative confocal images of retinal sections showing surviving RBPMS RGCs using the ocular drip drug delivery method in the HupA-treatment and control mouse groups. Scale bar, 20 μm. (B) Quantification of the RGC survival rate in (A) (unpaired t-test, ****p < 0.0001; n = 5 mice in each group, at least eight non-adjacent retinal sections per mouse). (C) Representative confocal images of retinal sections showing surviving RBPMS RGCs using vitreous cavity injection administration in the HupA-treatment and control mouse groups. Scale bar, 20 μm. (D) Quantification of the RGC survival rate in (C) (Data are represented as mean ± SEM, unpaired t-test, ****p < 0.0001; n = 5 mice in each group, at least eight non-adjacent retinal sections per mouse). [file Data_Sheet_2.ZIP › Raw date(Supplementary material)/Figure S4/image/PBS/11.tif]

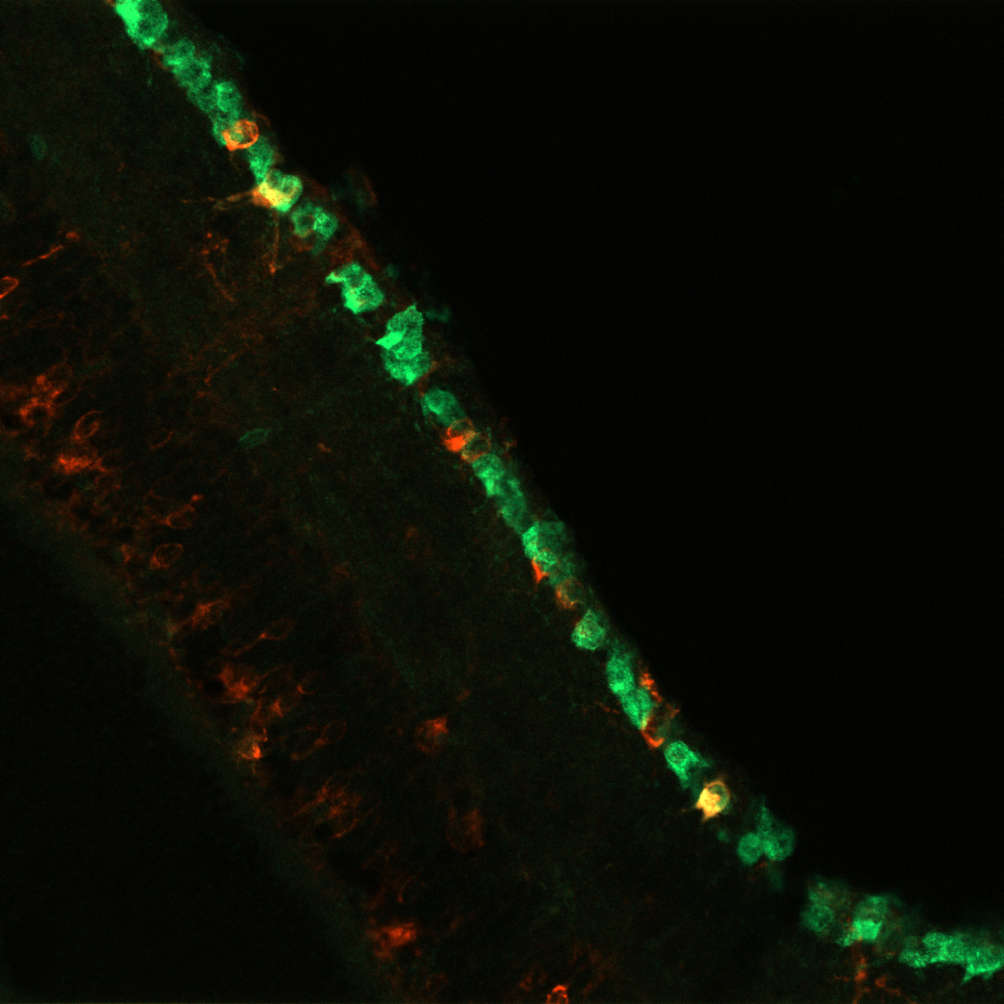

Supplement: Supplementary Figure 1 — Immunofluorescence staining of retinal sections at 14 days after ONC. (A) Representative confocal images of retinal sections showing surviving RBPMS RGCs using the ocular drip drug delivery method in the HupA-treatment and control mouse groups. Scale bar, 20 μm. (B) Quantification of the RGC survival rate in (A) (unpaired t-test, ****p < 0.0001; n = 5 mice in each group, at least eight non-adjacent retinal sections per mouse). (C) Representative confocal images of retinal sections showing surviving RBPMS RGCs using vitreous cavity injection administration in the HupA-treatment and control mouse groups. Scale bar, 20 μm. (D) Quantification of the RGC survival rate in (C) (Data are represented as mean ± SEM, unpaired t-test, ****p < 0.0001; n = 5 mice in each group, at least eight non-adjacent retinal sections per mouse). [file Data_Sheet_2.ZIP › Raw date(Supplementary material)/Figure S4/image/PBS/PBS.png]

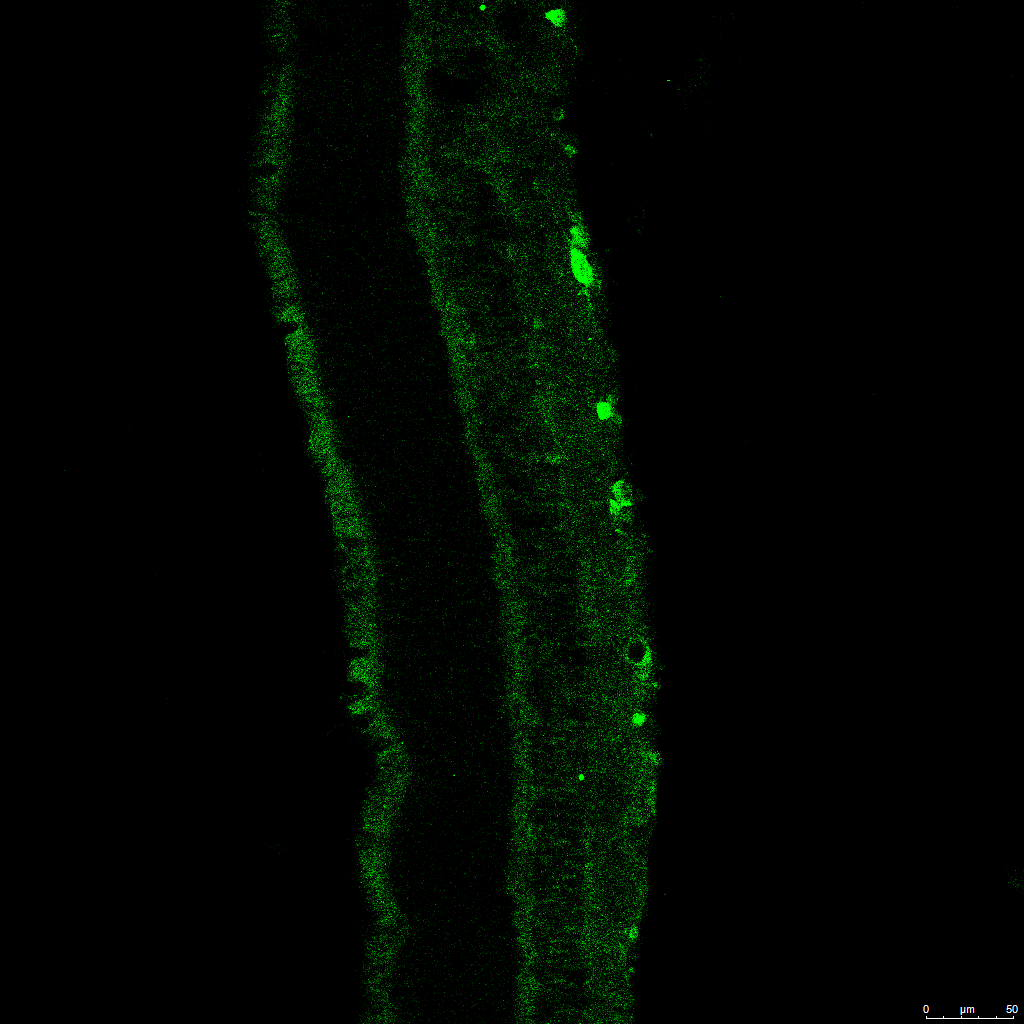

Supplement: Supplementary Figure 1 — Immunofluorescence staining of retinal sections at 14 days after ONC. (A) Representative confocal images of retinal sections showing surviving RBPMS RGCs using the ocular drip drug delivery method in the HupA-treatment and control mouse groups. Scale bar, 20 μm. (B) Quantification of the RGC survival rate in (A) (unpaired t-test, ****p < 0.0001; n = 5 mice in each group, at least eight non-adjacent retinal sections per mouse). (C) Representative confocal images of retinal sections showing surviving RBPMS RGCs using vitreous cavity injection administration in the HupA-treatment and control mouse groups. Scale bar, 20 μm. (D) Quantification of the RGC survival rate in (C) (Data are represented as mean ± SEM, unpaired t-test, ****p < 0.0001; n = 5 mice in each group, at least eight non-adjacent retinal sections per mouse). [file Data_Sheet_2.ZIP › Raw date(Supplementary material)/Figure S1/Image/Eye drop/con(PBS).tif]

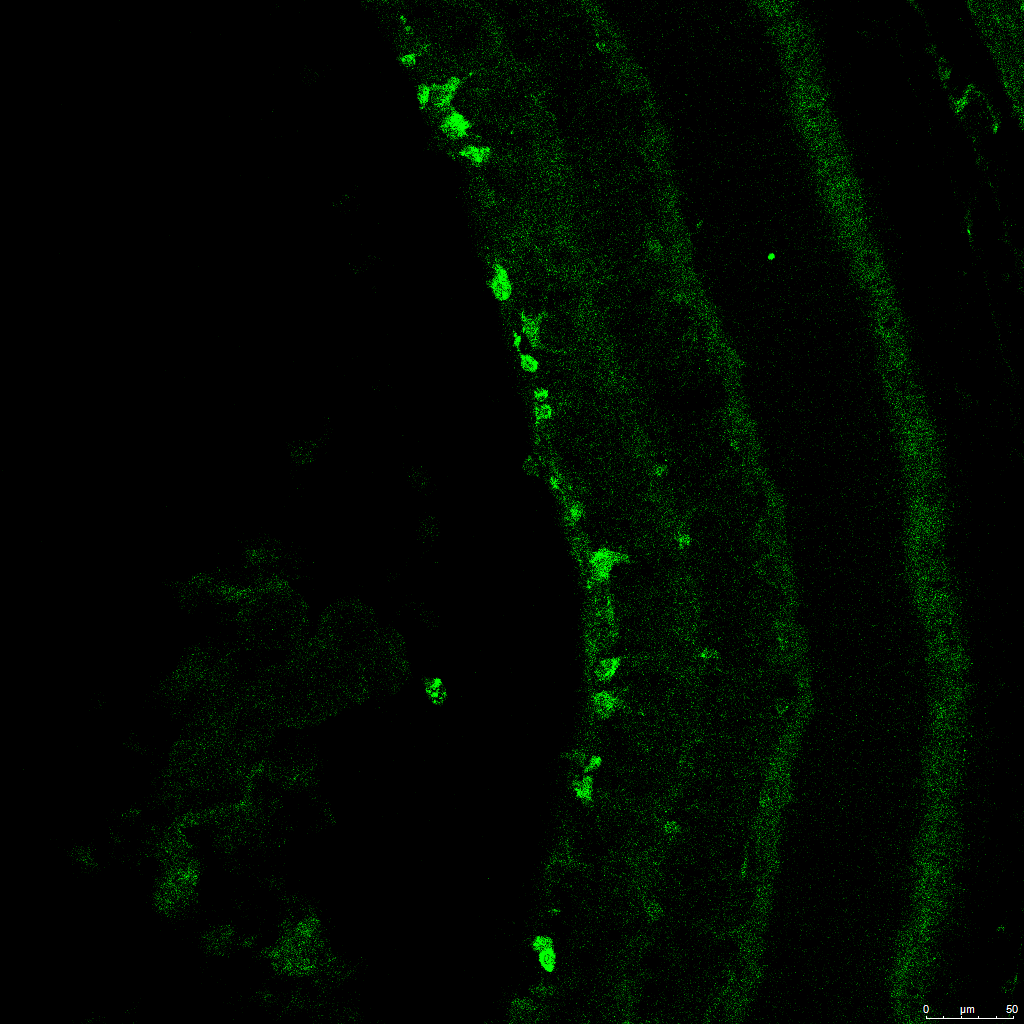

Supplement: Supplementary Figure 1 — Immunofluorescence staining of retinal sections at 14 days after ONC. (A) Representative confocal images of retinal sections showing surviving RBPMS RGCs using the ocular drip drug delivery method in the HupA-treatment and control mouse groups. Scale bar, 20 μm. (B) Quantification of the RGC survival rate in (A) (unpaired t-test, ****p < 0.0001; n = 5 mice in each group, at least eight non-adjacent retinal sections per mouse). (C) Representative confocal images of retinal sections showing surviving RBPMS RGCs using vitreous cavity injection administration in the HupA-treatment and control mouse groups. Scale bar, 20 μm. (D) Quantification of the RGC survival rate in (C) (Data are represented as mean ± SEM, unpaired t-test, ****p < 0.0001; n = 5 mice in each group, at least eight non-adjacent retinal sections per mouse). [file Data_Sheet_2.ZIP › Raw date(Supplementary material)/Figure S1/Image/Eye drop/HupA.tif]

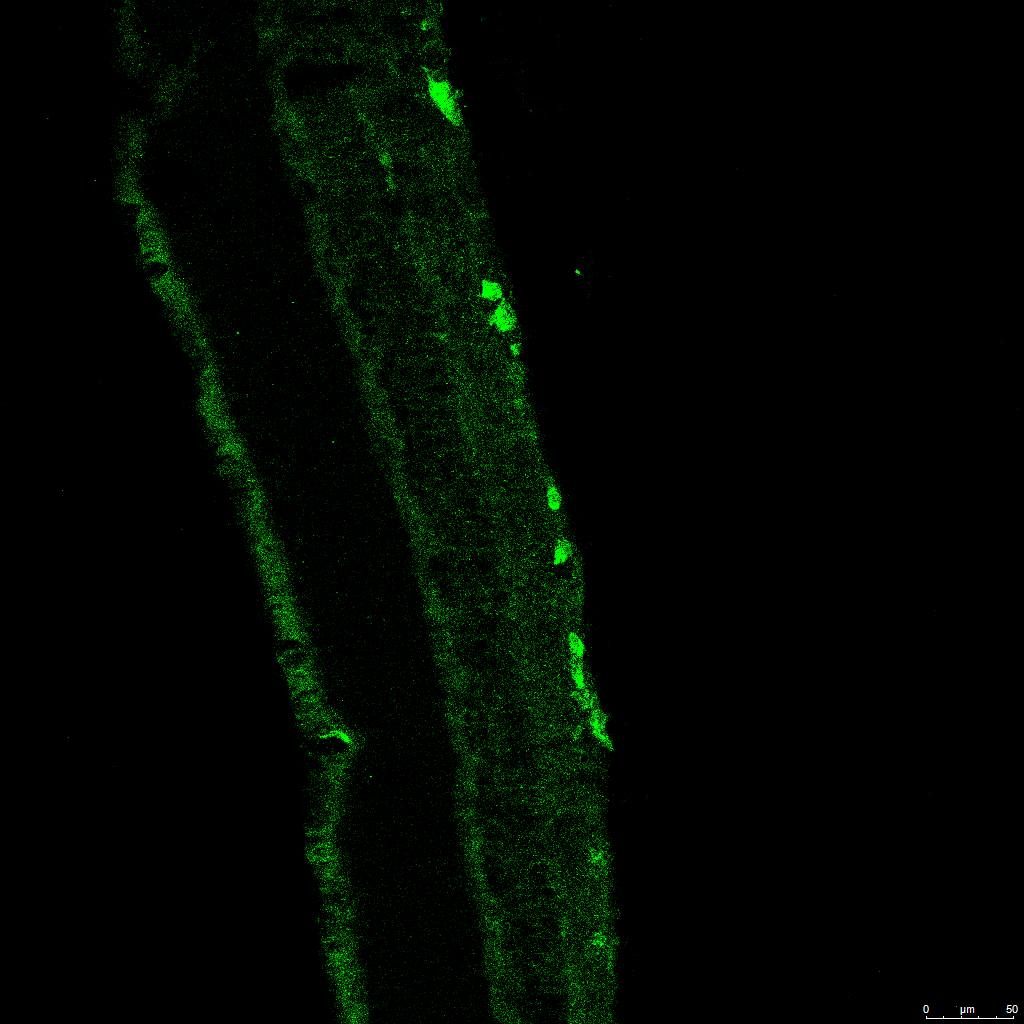

Supplement: Supplementary Figure 1 — Immunofluorescence staining of retinal sections at 14 days after ONC. (A) Representative confocal images of retinal sections showing surviving RBPMS RGCs using the ocular drip drug delivery method in the HupA-treatment and control mouse groups. Scale bar, 20 μm. (B) Quantification of the RGC survival rate in (A) (unpaired t-test, ****p < 0.0001; n = 5 mice in each group, at least eight non-adjacent retinal sections per mouse). (C) Representative confocal images of retinal sections showing surviving RBPMS RGCs using vitreous cavity injection administration in the HupA-treatment and control mouse groups. Scale bar, 20 μm. (D) Quantification of the RGC survival rate in (C) (Data are represented as mean ± SEM, unpaired t-test, ****p < 0.0001; n = 5 mice in each group, at least eight non-adjacent retinal sections per mouse). [file Data_Sheet_2.ZIP › Raw date(Supplementary material)/Figure S1/Image/Injection/con(PBS).tif]

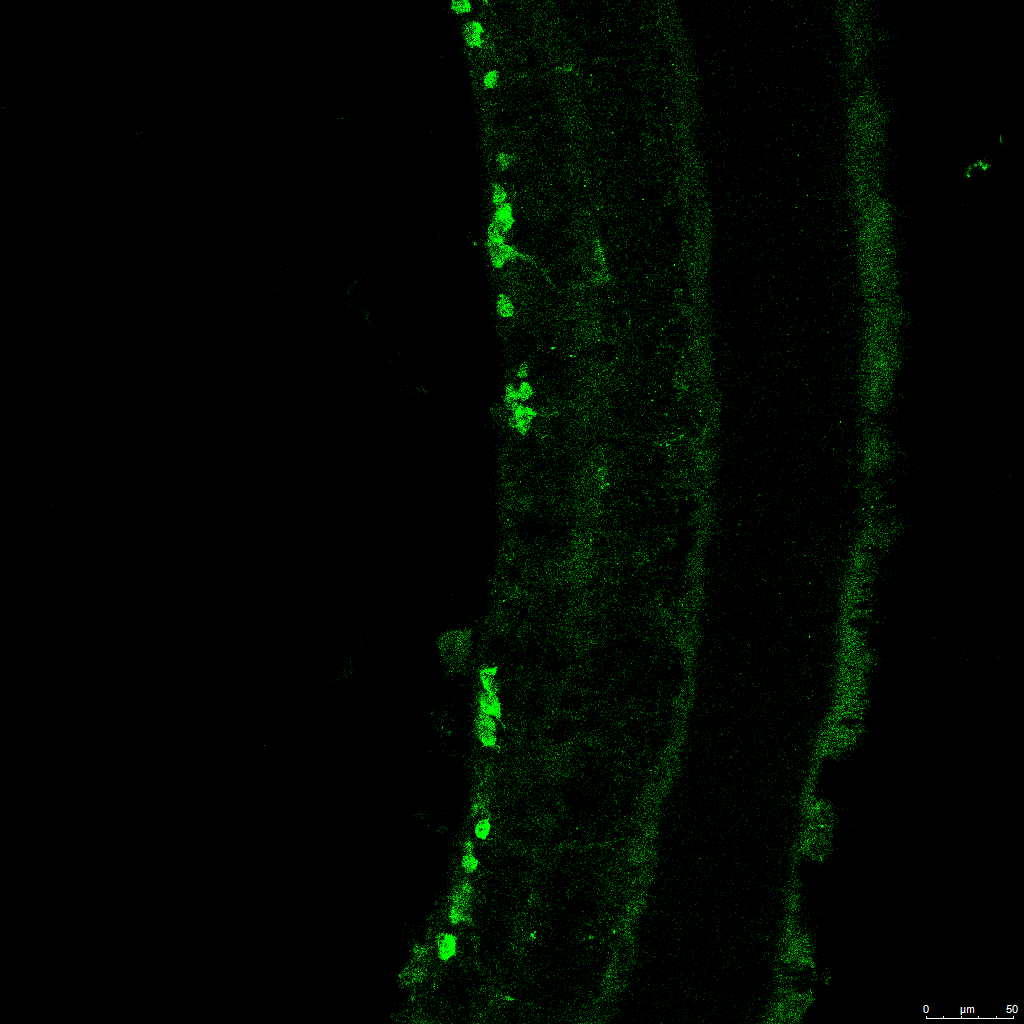

Supplement: Supplementary Figure 1 — Immunofluorescence staining of retinal sections at 14 days after ONC. (A) Representative confocal images of retinal sections showing surviving RBPMS RGCs using the ocular drip drug delivery method in the HupA-treatment and control mouse groups. Scale bar, 20 μm. (B) Quantification of the RGC survival rate in (A) (unpaired t-test, ****p < 0.0001; n = 5 mice in each group, at least eight non-adjacent retinal sections per mouse). (C) Representative confocal images of retinal sections showing surviving RBPMS RGCs using vitreous cavity injection administration in the HupA-treatment and control mouse groups. Scale bar, 20 μm. (D) Quantification of the RGC survival rate in (C) (Data are represented as mean ± SEM, unpaired t-test, ****p < 0.0001; n = 5 mice in each group, at least eight non-adjacent retinal sections per mouse). [file Data_Sheet_2.ZIP › Raw date(Supplementary material)/Figure S1/Image/Injection/HupA.tif]
